# Signal Processing in fNIRS: A Case for the Removal of Systemic Activity for Single Trial Data

**DOI:** 10.3389/fnhum.2019.00331

**Published:** 2019-09-24

**Authors:** Franziska Klein, Cornelia Kranczioch

**Affiliations:** ^1^Neurocognition and Functional Neurorehabilitation Group, Neuropsychology Lab, Department of Psychology, Faculty of Medicine and Health Science, University of Oldenburg, Oldenburg, Germany; ^2^Research Center Neurosensory Science, University of Oldenburg, Oldenburg, Germany

**Keywords:** fNIRS, EMG, neurofeedback, BCI, motor imagery, single trial analysis, signal improvement, global component removal

## Abstract

Researchers using functional near infrared spectroscopy (fNIRS) are increasingly aware of the problem that conventional filtering methods do not eliminate systemic noise at frequencies overlapping with the task frequency. This is a problem when signals are averaged for analysis, even more so when single trial data are used as in online neurofeedback or BCI applications where insufficiently preprocessed data means feeding back noise instead of brain activity or when looking for brain-behavior relationships on a trial-by-trial basis. For removing this task-related noise statistical approaches have been proposed. Yet as evidence is lacking on how these approaches perform on independent data, choosing one approach over another can be difficult. Here signal quality at the single trial level was considered together with statistical effects to inform this choice. Compared were conventional band-pass filtering and wavelet minimum description length detrending and the combination of both with a more elaborate, published preprocessing approach for a motor execution—motor imagery data set. Temporal consistency between Δ[*HbO*] and Δ[*HbR*] and two measures of the spatial specificity of signals that are proposed here served as measures of data quality. Both improved strongly for the combinationed preprocessing approaches. Statistical effects showed a strong tendency toward getting smaller for the combined approaches. This underlines the importance to adequately deal with noise in fNIRS recordings and demonstrates how the quality of statistical correction approaches can be estimated.

## 1. Introduction

The functional near infrared spectroscopy (fNIRS) signal is intended to measure task-related cortical hemodynamic responses. It typically also comprises physiological noise such as heart beat (1–1.5 Hz), respiration (0.2–0.5 Hz) and low frequency content resulting from blood pressure fluctuations (Mayer waves; 0.1 Hz) (Naseer and Hong, 2015; Kamran et al., 2016). Several methods are available to remove these systemic, non-evoked components (Jang et al., 2009; Zhang et al., 2013; Naseer and Hong, 2015; Kamran et al., 2016). The most widely used method is band-pass filtering with cut-off frequencies of approximately 0.01–0.9 Hz (Naseer and Hong, 2015; Kamran et al., 2016). However, depending on the context of an experiment (e.g., online vs. offline) filter types [e.g., finite impulse response (FIR) vs. infinite filter response (IIR); Pinti et al., 2018] and cutoff frequencies differ substantially (e.g, offline filter with cutoff frequencies of [0.01, 0.09] Hz as in Pinti et al., 2018 vs. online filter with cutoff frequencies of [0.01, 1.5] Hz as in Kober et al., 2014). Since the exact frequency characteristics of the systemic components are unknown and might differ between subjects, band-pass filtering might not eliminate all frequencies related to this physiological contamination (Duan et al., 2018). One possible approach to overcome this limitation might be the application of wavelet filters since in this context it is not relevant to know the exact frequency components (Jang et al., 2009; Duan et al., 2018).

In addition to the non-evoked systemic noise signals, another noise-component is task-dependent cerebral and extracerebral hemodynamic activity. This noise signal component is present in the signal due to near infrared (NIR) light passing cerebral and highly vascularized extracerebral layers, that is scalp and skull tissue, from a light source to a detector twice (Leff et al., 2011; Scholkmann et al., 2014; Tak and Ye, 2014; Brigadoi and Cooper, 2015; Tachtsidis and Scholkmann, 2016; Zhang et al., 2016; Pfeifer et al., 2018). During this journey the NIR light is contaminated by changes in the hemodynamic signals originating in these layers, mainly from the scalp veins (Takahashi et al., 2011; Kirilina et al., 2012). This noise component, consisting of evoked systemic cerebral and extracerebral components, here referred to as global component (GC), is considerably more problematic to remove than the non-evoked systemic noise because it is not constantly distributed over the head but varies from channel to channel (Gagnon et al., 2012). Moreover, it comprises the same task-related frequency range as the brain-related neuronal signal and can therefore not be removed by any filtering method. Although awareness for this fNIRS-specific problem is rising (Scholkmann et al., 2014; Tachtsidis and Scholkmann, 2016; Zhang et al., 2016; Pfeifer et al., 2018), to date most researchers do not correct for the GC and in consequence many of the reported experimental fNIRS effects are at risk of being inflated by artifacts (Brigadoi and Cooper, 2015; Pfeifer et al., 2018). In cases in which single trial data are used, the danger of quantifying artifacts is even more severe as here artifacts not properly removed by the standard filters will have a much stronger impact. Examples are neurofeedback or brain-computer interface (BCI) setups or studies aiming to demonstrate a brain-behavior relationship at the single trial level.

The use of additional short-distance channels (0.5–1 cm) is by now considered the most efficient method to remove the extracerebral component of the GC from the hemodynmaic fNIRS data but most up-and-running fNIRS systems are not equipped with the necessary hardware yet (Saager and Berger, 2005; Brigadoi and Cooper, 2015; Tachtsidis and Scholkmann, 2016; Nambu et al., 2017; Yücel et al., 2017; Pfeifer et al., 2018). Alternatives are a number of statistical correction methods, that however do not distinguish between the cerebral and the extracerebral aspect of the GC (Scholkmann et al., 2014; Tachtsidis and Scholkmann, 2016). Little is known of how these methods affect signals and statistical results in independent data sets (Zhang et al., 2005, 2016; Kohno et al., 2007; Santosa et al., 2013; Pfeifer et al., 2018).

Pfeifer et al. (2018) compared different approaches two of which included a self-implemented correction by means of a global regression or a unilateral regression. They found major differences between the averaged data in the tested approaches and also in the corresponding statistical results which emphasizes the extensive impact the GC might have on a study's results and corresponding conclusions.

This study continues this line of research with a particular focus on single trial data quality. Aim of the study was to quantify the impact of published preprocessing approaches on single trial data and statistical outcome of an independent data set. In a first step, the conventional band-pass filter was compared with a wavelet minimum description length (MDL) detrending filter (implemented in NIRS_SPM; Jang et al., 2009). The latter was expected to provide a superior removal of the non-evoked systemic noise from the fNIRS signal as compared to band-pass filtering. In a second step, to additionally remove the GC from the signal, both the band-pass filter and the wavelet filter were combined with an approach based on singular value decomposition (SVD) and a Gaussian kernel smoother (Zhang et al., 2016). These methods were selected because they can be easily implemented in an online neurofeedback or BCI experiment, yet several more exist, also regarding the removal of the GC (Zhang et al., 2005, 2016; Kohno et al., 2007; Santosa et al., 2013; Pfeifer et al., 2018).

Across the four approaches it was expected that the respectively more elaborate approaches, that is, each filtering method in combination with the SVD and Gaussian kernel method, would result in a higher signal quality as compared to the filtering methods alone. Moreover, it was expected that the task signal would be more spatially specific after applying the spatial filter in addition as compared to either the band-pass or the wavelet filter alone. Higher spatial specificity would be reflected in lower correlations between channels and more clearly defined activation patterns. These differences in signal quality were predicted to affect experimental effect sizes in the data set with a decrease from band-pass filtering to the wavelet filter to the combination of the filtering methods and global component removal.

## 2. Methods

### 2.1. Subjects

The analyzed data was originally collected as part of a fNIRS-based motor imagery (MI) neurofeedback group comparison study. Data from the different neurofeedback groups were pooled for the present analysis (for details see section 2 in the Supplementary Material). In total, 50 data sets [27 females, 23 males; mean (± SD) age: 24.1 (±2.78) years, ranging from 19 to 30 years] were included. Another ten subjects were excluded based on their electromyography (EMG) data (for details see section 3 in Supplementary Material). Handedness was assessed by means of the Edinburgh Handedness Inventory (EHI; Oldfield, 1971). All participants were right handed with a mean laterality quotient of 84.75 ± 17.33 (mean ± SD). Participants were recruited by way of the virtual platform of the University of Oldenburg. Only participants without a history of psychological and neurological disorders, with normal or corrected-to-normal vision and without any experience in piano playing were included. After explanation of the study, all subjects gave written informed consent. Participants were paid 8 €/h as reimbursement. The study was approved by the Ethics Committee of the University of Oldenburg.

### 2.2. Experimental Design

**Figure 1:**
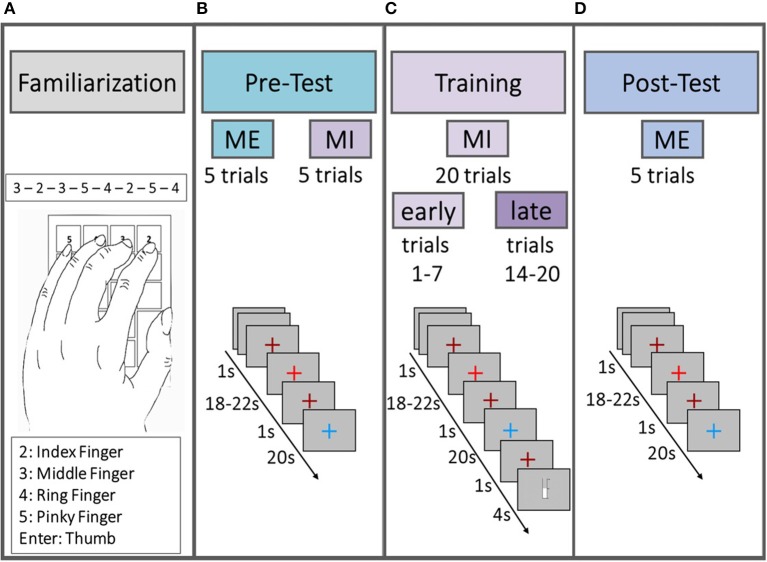
Schematic illustration of the experimental design. All subjects started with a familiarization phase **(A)** in which they memorized the sequence, followed by the pre-test **(B)**, consisting of five trials ME and five trials MI. Afterwards subjects performed 20 trials of MI training **(C)** and at the end a post-test **(D)** was performed, consisting of five trials of ME. The first seven trials in the MI training session **(C)** were defined as “early” trials and the last seven trials as “late” trials in order to statistically test for a “time on task” effect within the MI data.

Figure 1 illustrates the structure of the experimental design. Each subject participated in one experimental session consisting of familiarization phase, pre-test [motor execution (ME) and MI], training session (MI) and post-test (ME) that together lasted no longer than 45 min. During the session, subjects sat comfortably in a chair with their arms on the chair's armrests in a sound-shielded booth. Participants sat in front of a computer monitor at a distance of approximately 175 cm. The motor task was a sequential 8-position finger-tapping task performed with the left hand. The task was semi self-paced in that the duration of the tapping period was fixed, but not the speed with which participants tapped. Participants memorized the fixed sequence of numbers (3-2-3-5-4-2-5-4) during the familiarization phase.

In the ME parts of the pre-test and the post-test phases participants physically performed the motor task as often as possible in five 20 s trials. In the MI parts of the pre-test and the training phase, participants were instructed to perform the finger-tapping task as often as possible for five (pre-test phase) or 20 (training phase) 20 s trials using kinesthetic motor imagery. Participants were asked not to move their hands throughout the MI parts. In the training phase participants belonging to one of the feedback groups (cf. section 2 in Supplementary Material for details) were additionally instructed to increase a thermometer bar from trial to trial by trying to intensify the imagination of the motor task. Participants of the non-feedback group saw a comparable bar to keep visual input as similar as possible across groups. Trial structure was identical for MI and ME trials. A trial started with a rest stage lasting for 18–22 s (pseudo-randomized) indicated by a bright red fixation cross (cf. Figures 1B–D). The bright red fixation cross was preceded and followed by a 1 s dark red fixation cross indicating an upcoming change between trial stages. The subsequent task stage lasted for 20 s and was indicated by a blue fixation cross. In the training session the task stage was followed by an 1 s dark red fixation cross and either a visual feedback in shape of a thermometer filling up from the bottom according to the achieved change in hemoglobin concentration or a thermometer displaying the passing of time till the next trial would start. For a more detailed description of the experimental design including neurofeedback generation see section 2 in Supplementary Material.

### 2.3. Data Recording

#### 2.3.1. Electromyography (EMG) and Electroencephalography (EEG)

Electromyography (EMG) data was recorded from the extensor digitorum communis muscle on both arms during the whole experiment. To this end two electrodes were placed on each arm, resulting in two bipolar channels. A ground electrode was attached to the processus styloideus ulnae (see Figure S1 in Supplementary Material). EMG was recorded with a BrainVision Recorder (version 1.10) using a BrainAmp DC Amplifier (BrainProducts, Gilching, Germany). The sampling rate was 1 kHz with online filtering between 0.1 Hz and 250 Hz. The EMG data served as a control variable for the offline analysis only. All EMG-contaminated MI trials were removed from analysis (for details see section 3 in Supplementary Material).

In addition to the fNIRS data, EEG data was simultaneously recorded from 32 Ag/AECl electrodes placed together with the NIRS optodes in an elastic cap (EasyCap, Herrsching, Germany) according to the international 10–20 system. EEG data are of no relevance for the present study and will not be considered further.

#### 2.3.2. Functional Near Infrared Spectroscopy (fNIRS)

**Figure 2:**
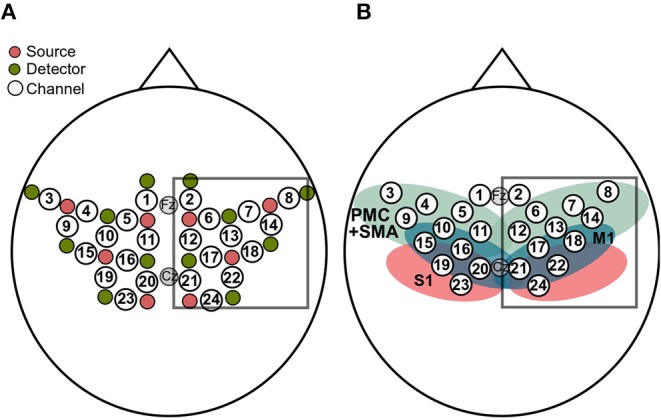
**(A)** The eight sources (pink) and the 12 detectors (green) resulted in 24 channels (white). Analyses were restricted to channels covering the right hemisphere (box). **(B)** Sensorimotor cortical regions covered by the layout, approximated by means of the fOLD toolbox (Zimeo Morais et al., 2018), together with the fNIRS channels. The regions include primary motor cortex (M1; blue), premotor cortex and supplementary motor areas (PMC+SMA; green) and the primary somatosensory cortex (S1; red). As in **(A)**, the box contains the channels used for analyses.

FNIRS data was recorded with a NIRScout 816 device (NIRStar 14, NIRx Medizintechnik GmbH, Berlin, Germany). As visualized in Figure 2, the eight LED sources (intensity 5 mW/wavelength) and the 12 detectors covered the premotor cortex (PMC), primary motor areas (M1), supplementary motor areas (SMA), and somatosensory areas (S1) of both hemispheres (approximated with the fOLD toolbox; Zimeo Morais et al., 2018). The NIRS optodes were placed according to the international 10–20 system in a custom-made cap together with the EEG electrodes. The Cz position was used as a marker for correctly positioning the cap. The optodes were attached to the cap with spring-loaded grommets (NIRx Medizintechnik GmbH, Berlin, Germany) which reduce optode movement and, which improve the contact between optode and skull. The distance between a source and a neighboring detector was approx. 3 cm and each of these source-detector pairs gave rise to one channel. This layout resulted in a total of 24 channels. The sources emitted NIR light at wavelengths 760 and 850 nm. Light intensity at the detectors was sampled with a sampling rate of 7.81 Hz.

## 3. Data Processing and Statistical Analysis

### 3.1. Data Preprocessing

For offline preprocessing using the band-pass filter, fNIRS data was imported via the MATLAB-based nilab2 toolbox (Rev. 2.0, NIRx Medizintechnik GmbH, Berlin, Germany) using MATLAB 2012a (The MathWorks Inc., Natick, MA, USA). To allow for the data to be preprocessed with the wavelet MDL detrending approach (Jang et al., 2009) data was imported via the NIRS_SPM toolbox (Version 4; Ye et al., 2009).

Raw data were then transformed into concentration changes of Δ[*HbO*] and Δ[*HbR*] by means of the modified Beer-Lambert law (mBLL; *DPF* = [5.98, 7.15] and ϵ = [2.5264, 1.7986;1.4866, 3.8437]). Data were preprocessed with either conventional band-pass filtering, wavelet MDL detrending, or a combination of either approach with the global component removal.

### 3.2. Conventional Band-Pass Filter (BPF)

In the context of a general linear model (GLM) framework it is recommended to apply a finite impulse response (FIR) filter with very high filter orders (i.e., > 1, 000; Pinti et al., 2018). Since the context of the present analyses differs, that is, the prospective application within a (online) neurofeedback or BCI experiment, a FIR filter with such a high filter order (i.e., the filter length minus one data point) is not applicable without a very long baseline (e.g., around 1, 000/8*s*^−1^ = 125*s* with a sampling rate of approx. 8 Hz). In addition, MATLAB-based filter functions such as *filtfilt*() require data lengths of at least three times the filter order. Therefore, an infinite impulse response (IIR) filter was used in the present study in order to have a better comparison to the outcome of an online preprocessing version.

Although a broader frequency window of the band-pass filter was applied in the online version of this experiment (i.e., [0.01, 0.7] Hz; cf. section 3 in Supplementary Material) the guidelines regarding the cutoff frequency window of Pinti et al. (2018) was followed. That is, for either the conventional band-pass filtering (BPF) approach and its combination with the global component removal (BPF+GCR) the data of the Δ[*HbO*] and Δ[*HbR*] was firstly low-pass filtered with a third order Butterworth filter (0.09 Hz) and afterwards high-pass filtered by means of a first order Butterworth filter (0.01 Hz). The filters were applied using MATLAB-based functions [i.e., *butter*() and *filtfilt*()].

### 3.3. Wavelet Filter (WLF)

In order to eliminate the non-evoked systemic components from the hemoglobin signal (i.e., respiration, blood pressure) a wavelet MDL detrending method was applied (Jang et al., 2009) which is implemented in the MATLAB-based NIRS_SPM toolbox (Version 4; Ye et al., 2009). This wavelet filter (WLF) has some advantages over conventional band-pass filtering. For example, it is possible to remove the frequencies due to physiological noise and fast varying trends, which can not be removed by conventional filtering because otherwise parts of the hemodynamic signal would be removed (Jang et al., 2009). The method was originally developed for fMRI analysis and later adapted for fNIRS data analysis. It is based on the discrete wavelet transform and it is used to decompose the fNIRS data into global trend (i.e., global drift), hemodynamic signals and uncorrelated noise at distinct scales (Jang et al., 2009). For the decomposition, the data is firstly temporally smoothed using a canonical hemodynamic response function (HRF) (Friston et al., 2000), whereupon a wavelet MDL detrending algorithm (Jang et al., 2009) removes the global trend from the signal. In the algorithm of Jang et al. (2009), this global trend is modeled within the general linear model (GLM):

(1) $$\begin{array}{r} Y=X\beta +\epsilon +\theta \end{array}$$

where the fNIRS signal is indicated by *Y*, the design matrix by *X*, β describes the weights and ϵ the residuals. The new term denoted by θ characterizes the additional global drift for each wavelength and at each channel location separately. Therefore, a modified GLM is derived for each signal type *HbX* as follows:

(2) $$\begin{array}{r} Y_{HbX}=X_{HbX}\beta_{HbX}+\epsilon_{HbX}+\theta_{HbX} \end{array}$$

Then, a discrete wavelet transform is used to separate the global trend θ_*HbX*_ into wavelets. In comparison to the infinite sine and cosine waves used in Fourier analysis, wavelets are temporally limited “mini waves” used to represent other functions. Fourier analysis techniques use sine and cosine functions which are defined by frequency and not by time. A small change in frequency produces changes in the whole time domain, whereas wavelets are local in frequency and time (Vidakovic and Müller, 1994). To denoise or filter the data by means of wavelets some coefficients act as filters and others correspond to details in the data set. The idea is to set all coefficients below a specified threshold to zero before using them for reconstruction of the data in an inverse wavelet transform. This way of filtering is favored because it denoises the signal without removing sharp structures and details (Graps, 1995). Therefore, Jang et al. (2009) implemented a Cohen–Daubechies–Feauveau (CDF) 9/7 wavelet. For its usage in fMRI exists an implicit assumption that all wavelet coefficients at the same scale are simultaneously either all zero or all non-zero (Jang et al., 2009). However, fNIRS data possesses a much higher number of data points and if the same assumption is considered it could lead to under- or overfitting. To avoid this, the MDL principle is additionally implemented. Simplified, this procedure is based on Occam's razor (i.e., select the answer with the fewest assumptions) and successively includes the descending ordered wavelet coefficients until a sufficient complexity is found (Jang et al., 2009).

### 3.4. Wavelet Filter and GC Removal (WLF+GCR) and Band-Pass Filter and GC Removal (BPF+GCR)

In addition to both the BPF and the WFL method, the GC was removed from the signal. One method for removing the GC was introduced by Zhang et al. (2016). This method is based on Gaussian spatial filtering and singular value decomposition (SVD). For implementing the method, the Montreal Neurological Institute (MNI) coordinates of each channel are required. The MNI coordinates for this data set were approximated by means of the ICBM-152 head model (ICBM 2009a Nonlinear Symmetric Atlas) with the NIRSite toolbox (Version 1, NIRx Medizintechnik GmbH, Berlin, Germany). The optodes were not digitized for any of the subjects, instead uniform coordinates according to the international 10–20 system (approximated by the fOLD toolbox; Zimeo Morais et al., 2018) were used. Although subject specific coordinates would have been more accurate, the distances between optodes are primarily important for this approach and should be comparable between subjects.

#### 3.4.1. Gaussian Spatial Filtering

To smooth the data with the Gaussian kernel channel distances are required. In order to calculate the curved distance between two channels (Fenn, 2001) the conversion from cartesian (*x, y, z*) into spherical coordinates (*r*, θ, φ) is necessary. The distance between two channels is then calculated by means of the arc length which is defined as the great-circle distance, i.e., the shortest distance between two points (channels) on a curved surface. To calculate this distance, information about the latitude and longitude of two channels, given by φ_1_, θ_1_ and φ_2_, θ_2_, is necessary, respectively. The central angle Δ*c* between the channels is then defined as:

(3) $$\begin{array}{r} \Delta c=\arccos\left(\sin\varphi_{1}\sin\varphi_{2}+\cos\varphi_{1}\cos\varphi_{2}\cos\left(\Delta \theta \right)\right) \end{array}$$

and distance *d* or arc length between these channels is then calculated as follows:

(4) $$\begin{array}{r} d=r\Delta c \end{array}$$

The distances between all channels are stored in a *n* × *n* distance matrix *D* which is applied within the Gaussian filter defined by:

(5) $$\begin{array}{r} G\left(D\right)=e^{\frac{-D^{2}}{2\sigma^{2}}} \end{array}$$

A Gaussian filter is a two-dimensional kernel smoother mainly applied to remove detail and noise from a data set. The variable σ represents the width of the kernel. The authors of the approach suggested σ = 50° because the width of the kernel should be on the one hand greater than the cortical neuronal activation and on the other hand smaller than the GC. In a later paper, enhanced results were reported by using a σ of 48° which is also applied in this analysis (Zhang et al., 2017).

#### 3.4.2. Singular Value Decomposition

The fNIRS signal over all channels is represented by an *m* × *n* matrix *S* where *n* specifies the number of channels and *m* the number of data points. A singular value decomposition (SVD) is basically a generalization of the eigendecomposition to non-symmetric matrices. The SVD decomposes the matrix *S* into three matrices:

(6) $$\begin{array}{r} S=U\Sigma V^{T} \end{array}$$

where *U* is an *m* × *n* matrix consisting of left singular vectors and represents the temporal waveform of the *n* channels (Harner, 1990; Zhang et al., 2016). The columns represent the principal features in descending order starting with the first column which is the highly correlated (most global) feature of the original data (Harner, 1990). Σ is a *n* × *n* diagonal matrix with *n* non-negative singular values as diagonal elements. The singular values correspond to the square root of the variance of each spatio-temporal feature which is the same as the square root of the eigenvalues and are arranged in Σ in descending order (Harner, 1990). *V*^*T*^ is the transpose of a *n* × *n* matrix and consists of the right singular vectors containing the spatial information of the channels corresponding to the temporal information of each vector in *U* (Harner, 1990; Zhang et al., 2016). Since the matrix *V* contains the spatial information of the fNIRS data, the next step toward the GC removal is the smoothing of *V* by means of the Gaussian filter. This kernel smoothing is done by a discrete convolution of *V*, consisting of vectors *v*_*i*_, and the Gaussian kernel *G*:

(7) $$\begin{array}{r} v_{i}^{*}=v_{i}*G \end{array}$$

The resulting matrix V^*^, consisting of the vectors $v_{i}^{*}$, contains only the spatial information of the GC, because the convolution eliminates the localized neuronal pattern (Zhang et al., 2016). To reconstruct the waveforms of the GC of each channel the matrix *V* is replaced by the matrix V^*^ in the SVD formula:

(8) $$\begin{array}{r} S_{Global}=U\Sigma V^{*} \end{array}$$

The difference between *S*_*Global*_ and *S* results in a matrix containing only data related to neuronal activity:

(9) $$\begin{array}{r} S_{Neuronal}=S-S_{Global} \end{array}$$

### 3.5. Channel Selection and Mean Value Calculation

**Figure 3:**
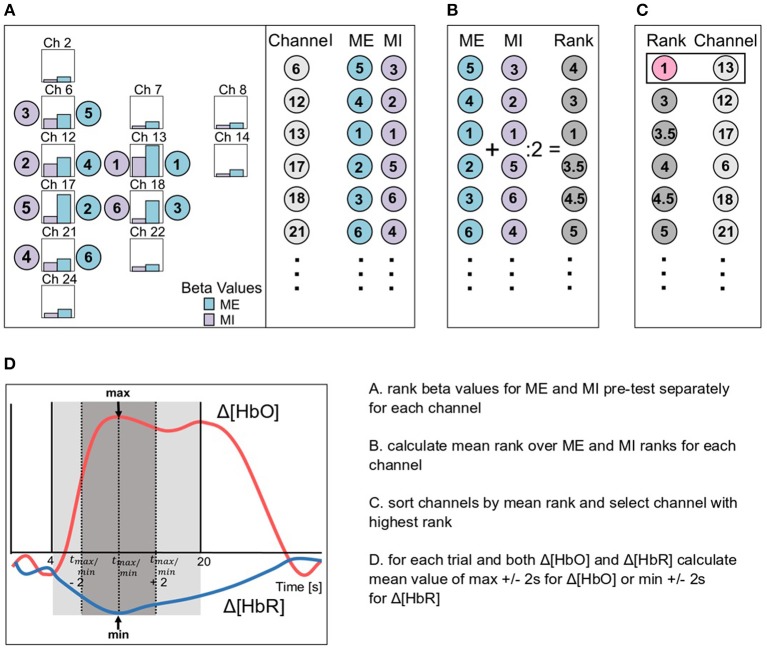
Schematic illustration of the channel selection based on the beta values (bars in **A**) for ME (blue) and MI (violet) of the pre-test phases. **(A)** Firstly, the channels were ordered due to the ranking of the beta values for ME and MI data separately, then **(B)** a second ranking was performed based on the mean rank of ME and MI ranks and finally **(C)**, the channel with the best rank was selected (channel 13 in the example). In **(D)** the mean value calculation is exemplary visualized for both Δ[*HbO*] (pink) and Δ[*HbR*] (blue) data. Therefore, for each trial a mean value over the peak ±2 s was calculated.

Once data were preprocessed with either the BPF, the WLF, the BPF+GCR or the WLF+GCR approach, GLMs were fitted to the cleaned data using a Boynton canonical HRF (6 s delay). GLMs were fitted using the nilab2 toolbox (Rev. 2.0, NIRx Medizintechnik GmbH, Berlin, Germany). Thereupon, all data was epoched (−5 to 30 s around stimulus onset) and baseline corrected (−5 to onset) using custom MATLAB-based code. All steps were applied to both Δ[*HbO*] and Δ[*HbR*]. Individual channel selection for statistical analysis was primarily based on the beta values derived from the GLM for the MI and ME pre-test phases and restricted to channels covering the right hemisphere, that is, the hemisphere contralateral to the active hand (cf. Figure 2). To select the individual channel, all contralateral channels were ranked according to their beta values separately for MI and ME (cf. Figure 3A). Then a second ranking was performed by averaging the ranks of ME and MI for each channel and the channel with the highest average rank was then chosen for further analyses (cf. Figures 3B,C). If there were two or more channels with the same average rank an additional criterion was applied. For this criterion, for each trial the mean value of the ±2 s window around the trials' peak was calculated (see Figure 3D). The mean values were then averaged separately for MI and ME trials. The resulting mean concentration changes for ME and MI were multiplied, and the channel with the largest product was selected for further analyses.

For the individually selected best channel, single trial mean values were extracted from a time window ranging from 4 to 20 s after stimulus onset. This window was selected because the hemodynamic response is not expected to peak before 4–6 s after stimulus onset due to the delayed nature of the signal and otherwise not task-related activity might influence the signal values used for statistics. Single trial mean concentration changes were derived as the mean value of the ±2 s window around the trial's peak. Single trial values were averaged separately for MI and ME trials.

### 3.6. Statistical Analyses

For all statistical analyses R (version 3.6.0 “Planting of a Tree”; R Core Team, 2017) together with RStudio (version 1.2.1335; RStudio Team, 2015) was used. The data was always visually checked for normally distributed residuals by means of a qq-plot. In order to correct for violations of sphericity (ANOVA) the Greenhouse-Geisser correction method was used. As *post-hoc* tests, Bonferroni corrected paired student's *t*-tests or pairwise *t-*tests (for parametric tests) as well as pairwise comparisons using Nemenyi multiple comparison test (for non-parametric tests) were applied. Additionally, test specific effect sizes were reported for all applied tests (i.e., Kendall's *W*, Cohen's *d* or generalized $\eta_{G}^{2}$).

#### 3.6.1. Temporal Consistency

To assess the quality of the neuronal signal *S*_*Neuronal*_, Zhang et al. (2016) took advantage of the assumption that Δ[*HbO*] and Δ[*HbR*] show a consistent spatial as well as a consistent temporal pattern and that both signal types show a linear relationship. They modeled the Δ[*HbO*] signal as a function of the Δ[*HbR*] signal of the channels covering the motor areas:

(10) $$\begin{array}{r} Oxy_{wave\;form}=\beta \cdot Deoxy_{wave\;form}+\epsilon \end{array}$$

where *Oxy*_*wave form*_ and *Deoxy*_*wave form*_ are vectors containing the grand average signal of the epoched data over all channels of interest with mean value removed. The β value describes the scalar to minimize the residual error ϵ. Zhang et al. (2016) suggested this error as a measure of temporal consistency and, this measure is predicted to decrease after GC removal. Additionally, the authors measured the spatial consistency by means of a similar model, and both measures showed significantly decreased residuals for the models fitted for *S*_*Neuronal*_ (Equation 6) as compared to the models fitted for the signal *S* (Equation 9) (Zhang et al., 2016). This reduced ϵ indicates an enhanced temporal relationship between Δ[*HbO*] and Δ[*HbR*], interpretable as resulting from a cleaner signal.

Temporal consistency was calculated as proposed by Zhang et al. (2016) for the different preprocessing approaches to judge preprocessing-related changes in data quality. In contrast to Zhang et al. (2016) no block averaged but single trial linear regression models were fitted separately for ME and MI data with Δ[*HbO*] and Δ[*HbR*] as the response variable and the explanatory variable (Equation 10). For all approaches, data for temporal consistency analysis were derived from the individual channels selected for the WLF+GCR preprocessed Δ[*HbO*] data because it was expected that this combination results in the cleanest signal and hence most accurate channel selection. For each subject and separately for ME and MI data, the residuals of the single trial regression models were extracted and a mean residual value was calculated. Note that for ME this average comprised all trials of pre- and post-test (ten trials in total) and for MI all trials of the training session after removal of the trials with significant EMG activity (17.72 ± 3.49 trials, ranging from 5 to 20 trials; for details of EMG analysis and EMG-based trial removal, see section 3 in Supplementary Material).

The mean of the single trial residuals were then used to test for differences in the temporal consistency of data derived with the four preprocessing approaches. Because the residuals of the data did not appear normally distributed a non-parametric repeated measures ANOVA (Friedman test) was conducted with the within-subject factor “approach” (BPF, WLF, BPF+GCR, WLF+GCR) and the mean residuals as response variable. Non-parametric tests are based on ranks which are in this case not informative, therefore in addition to mean ranks (± SEM), mean residuals (± SEM) are reported.

#### 3.6.2. Spatial Specificity

Spatial specificity refers to whether the signals measured with a given imaging technique are clearly defined in space or smeared, where a clearly defined signal would be the desirable situation. To quantify spatial specificity of Δ[*HbO*] and Δ[*HbR*], the mean of single trial correlation matrices and spatial variances were calculated and compared for the four preprocessing approaches. To this end, for each subject Spearman correlation matrices were calculated between the single trial hemodynamic data of all contralateral channels, separately for ME and MI as well as Δ[*HbO*] and Δ[*HbR*]. Then two analyses were conducted.

For the first analysis, for each subject the single trial correlation matrices were averaged for each condition (ME and MI), signal type (Δ[*HbO*] and Δ[*HbR*]) and preprocessing approach (BPF, WLF, BPF+GCR, and WLF+GCR), resulting in 16 correlation matrices per subject. Then, for each of the 16 matrix types a mean correlation matrix over all subjects was calculated and normalized (Fisher Z transformation). The “cortest” function (“psych” package version 1.8.12) was used to test for differences between matrices resulting from the four preprocessing approaches. Matrices were expected to differ between BPF, WLF, BPF+GCR, and WLF+GCR as a result of improved artifact removal and, consequently, a more clearly defined hemodynamic signal. A significant difference between matrices is an indication that spatial specificity differs between approaches, it is however not unequivocal in this regard. That is, it could result from a spatially more clearly defined signal, but also from a spatially unspecific, general shift in correlations for one approach. This was addressed in the second analysis.

For the second analysis, the variances over the triangular matrix of the single trial correlation matrices was calculated. With this approach, a spatially unspecific signal would result in a smaller variance than a signal clearly defined in space. On the other hand, a spatially unspecific, general shift of correlations would not reduce the variance. For each participant and preprocessing approach, single trial variances were then averaged for ME and MI and Δ[*HbO*] and Δ[*HbR*], respectively. These values were used in four non-parametric repeated measures ANOVAs (Friedman test), all with the within-subject factor “approach” (BPF, WLF, BPF+GCR, WLF+GCR). Mean variances were expected to increase from BPF to WLF to BPF+GCR to WLF+GCR. Mean ranks (± SEM) and mean residuals (± SEM) are reported for these analyses.

#### 3.6.3. Consequences for Experimental Outcomes

Removing artifacts from fNIRS data with more elaborate preprocessing pipelines has been shown to affect the outcome of a study (Pfeifer et al., 2018). To examine whether the same applies in the present study a 4 × 2 ANOVA with repeated measures was conducted for the within-subject factors “approach” (BPF, WLF, BPF+GCR, WLF+GCR) and “time on task” (pre, post [ME] or early, late [MI]), and the averaged single trial mean values of the concentration changes as response variable for ME and MI and signal type separately. With regard to the experimental factor “time on task” it was expected that concentrations would change from pre to post (ME) and from early to late (MI), reflecting practice effects in the finger tapping task. For ME, the levels of the “time on task” factor corresponded to the data collected in pre- and post-test phases respectively. In contrast, for MI the two levels of the “time on task” factor were defined as the first seven trials (early) and the last seven trials (late) out of a total 20 trials of the MI training session. Participants had to have at least two valid trials for each level (i.e., 2 out of 7) in order to be entered into the analysis. Due to the EMG-based epoch removal this criterion was not fulfilled in three out of 50 participants, yielding a sample of *N* = 47 for this analysis.

The actual number of trials entering the analysis was (mean ± SD) 6.38 ± 1.24 (range: 2–7) for MI early trials, and for MI late trials 6.40 ± 1.06 (range: 2–7).

## 4. Results

### 4.1. Temporal Consistency

In order to assess whether the temporal consistency between Δ[*HbO*] and Δ[*HbR*] differs between the preprocessing approaches, linear models were fitted for each trial for the individually selected channel (see section Temporal Consistency). Individual single trial residuals were averaged and Friedman tests with the within-subject factor “approach” (BPF, WLF, BPF+GCR, WLF+GCR) were conducted for ME and MI separately.

**Figure 4:**
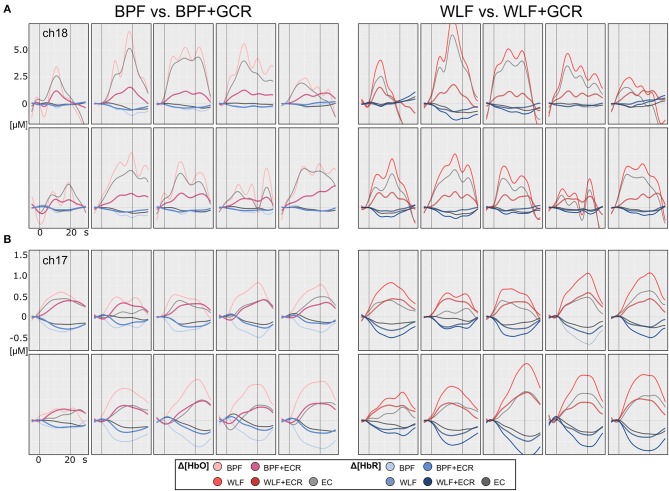
Motor execution single subject plots of two exemplary subjects **(A,B)** of Δ[*HbO*] and Δ[*HbR*] data. The subplots in the left column contain epoched ([−5, 30] s) and baseline corrected ([−5, 0] s) data of a single ME trials preprocessed by BPF and BPF+GCR approaches (BPF vs. BPF+GCR) and the subplots in the right column contain the data preprocessed by means of WLF and WLF+GCR approaches and the GC itself (WLF vs. WLF+GCR). For each subject, the first row contains the pre-test and the second row the post-test ME data.

**Figure 5:**
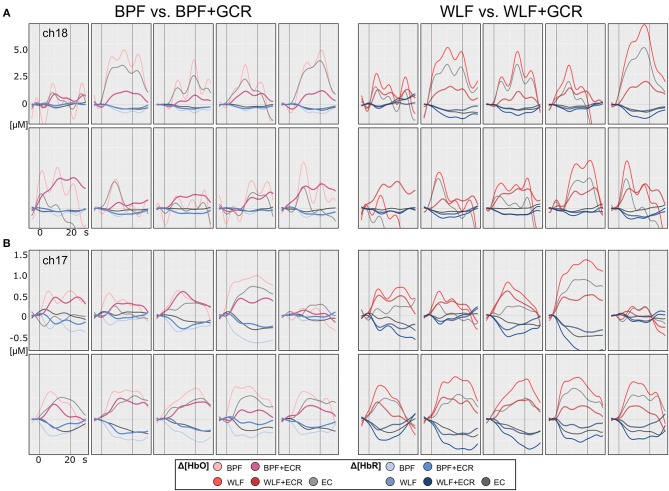
Motor imagery single subject plots of two exemplary subjects **(A,B)** of Δ[*HbO*] and Δ[*HbR*] data. The subplots in the left column contain epoched ([−5, 30] s) and baseline corrected ([−5, 0] s) data of a single MI trials preprocessed by BPF and BPF+GCR approaches (BPF vs. BPF+GCR) and the subplots in the right column contain the data preprocessed by means of WLF and WLF+GCR approaches and the GC itself (WLF vs. WLF+GCR). For both subjects for a better visualization only the first 10 trials of the training session after EMG correction are shown.

Figures 4, 5 illustrate the Δ[*HbO*] and Δ[*HbR*] data from two representative participants for ME (Figure 4) and MI (Figure 5) trials. Clearly, for both subjects the noise was highly reduced after applying the GCR approach in addition to each of the two filters in all trials. Furthermore, descriptively, after additionally removing the GC from the data the amplitudes of both Δ[*HbO*] and Δ[*HbR*] were greatly reduced as well as smoothed.

Results of the Friedman tests indicated for both MI and ME residuals a highly significant effect [ME: $\chi_{\left(3\right)}^{2}=146.62$, *p* < 0.00001, *W* = 0.89; MI: $\chi_{\left(3\right)}^{2}=135.96$, *p* < 0.00001, *W* = 0.87]. The subsequent pairwise comparisons using Nemenyi multiple comparison test resulted for all approaches and for both ME and MI in highly significant differences (*p* < 0.01).

Since the WLF+GCR approach was expected to result in the cleanest signal the channel selection was based on the data resulting from this approach. Against the expectations, the mean (± SEM) ranks and residuals were highest for WLF and lowest for BPF+GCR (cf. Table 1 for exact values), indicating that the highest temporal consistency resulted from the BPF+GCR approach. Hence, the analysis was repeated with the channel selection based on the BPF+GCR approach for which the same increments regarding the residuals were expected as found for the channel selection based on WLF+GCR.

**Table 1:** Temporal Consistency: Statistical results of the Friedman test and the *post-hoc* pairwise comparisons using Nemenyi multiple comparison test.

**Channel Selection based on WLF+GCR**										
**ME**: $\chi_{\left(3\right)}^{2}=146.62$, *p* < 0.00001, *W* = 0.89						**MI**: $\chi_{\left(3\right)}^{2}=135.96$, *p* < 0.00001, *W* = 0.87				
	**BPF**	**BPF +GCR**	**WLF**	**μ_*rank*_ ± SEM**	**μ_*res*_ ± SEM [μ*M***]	**BPF**	**BPF +GCR**	**WLF**	**μ_*rank*_ ± SEM**	**μ_*res*_ ± SEM [μ*M***]
BPF	–	–	–	125.40 ±6.73	1.06 ±0.08	–	–	–	126.04 ±7.10	0.77 ±0.05
BPF +GCR	*p* < 0.00001	–	–	58.38 ±6.51	0.52 ±0.05	*p* < 0.00001	–	–	56.94 ±5.97	0.36 ±0.03
WLF	*p*<0.001	*p* < 0.00001	–	143.70 ±6.04	1.30 ±0.10	*p*<0.01	*p* < 0.00001	–	143.58 ±6.43	0.93 ±0.06
WLF +GCR	*p*<0.01	*p*<0.001	*p* < 0.00001	74.52 ±6.86	0.62 ±0.05	*p*<0.01	*p*<0.001	*p* < 0.00001	75.44 ±6.48	0.44 ±0.03
**Channel Selection based on BPF+GCR**										
**ME**: $\chi_{\left(3\right)}^{2}=142.01$, *p* < 0.00001, *W* = 0.90						**MI**: $\chi_{\left(3\right)}^{2}=137.35$, *p* < 0.00001, *W* = 0.88				
	**BPF**	**BPF +GCR**	**WLF**	**μ_*rank*_ ± SEM**	**μ_*res*_ ±SEM [μ*M***]	**BPF**	**BPF +GCR**	**WLF**	**μ_*rank*_ ±SEM**	**μ_*res*_ ±SEM [μ*M***]
BPF	–	–	–	123.72 ±6.87	1.05 ±0.08	–	–	–	125.06 ±6.87	0.76 ±0.05
BPF +GCR	*p* < 0.00001	–	–	58.96 ±6.72	0.54 ±0.05	*p* < 0.00001	–	–	57.38 ±6.27	0.37 ±0.03
WLF	*p*<0.001	*p* < 0.00001	–	142.54 ±5.89	1.31 ±0.10	*p*<0.01	*p* < 0.00001	–	142.40 ±6.22	0.91 ±0.06
WLF +GCR	*p*<0.01	*p*<0.001	*p* < 0.00001	76.78 ±7.16	0.65 ±0.06	*p*<0.01	*p*<0.001	*p* < 0.00001	77.16 ±6.74	0.45 ±0.03

*Additionally, the mean (± SEM) ranks and residuals are represented for each method and ME and MI data*.

This was confirmed by the results of the Friedman test which showed also a highly significant effect for both ME [$\chi_{\left(3\right)}^{2}\;=\;142.01$, *p* < 0.00001, *W* = 0.90] and MI [$\chi_{\left(3\right)}^{2}=137.35$, *p* < 0.00001, *W* = 0.88] data. The *post-hoc* tests showed highly significant differences in all comparisons (*p* < 0.01). As reported in Table 1 the highest residuals resulted from the WLF approach, followed by BPF and decreased further from WLF+GCR to BPF+GCR approach, indicating the strongest temporal consistency resulting from the BPF+GCR method.

### 4.2. Spatial Specificity

To examine whether the more elaborate approaches resulted in a higher spatial specificity, single trial Spearman correlations were computed between all contralateral channels separately for Δ[*HbO*] and Δ[*HbR*], ME and MI and the four preprocessing approaches. Afterwards, mean correlation matrices were calculated, normalized and used to test for differences between methods. Secondly, the variances of the single trial correlation matrices were averaged. These values entered the Friedman tests with “approach” (BPF, WLF, BPF+GCR, WLF+GCR) as within-subject factor.

**Figure 6:**
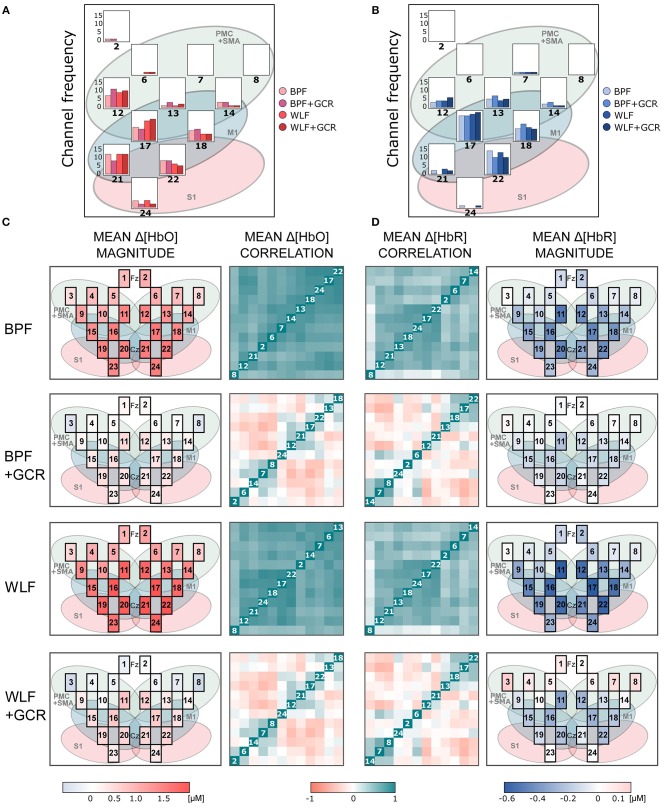
**(A,B)** Frequency of selected channels based on mean ranking of the ME and MI beta values resulting from the pre-test of **(A)** Δ[*HbO*] and **(B)** Δ[*HbR*] data. **(C,D)** Mean correlation matrices (non-normalized) and channel maps of mean magnitudes of the ME **(C)** Δ[*HbO*] and **(D)** Δ[*HbR*] data. Each row corresponds to one of the preprocessing approaches. For both signal types only WLF+GCR and BPF+GCR approaches resulted in a higher spatial specificity indicated by both mean correlation matrices and mean magnitudes. The white numbers in the diagonal correspond to the channel number. Note that the order of the channels might differ between matrices due to the hierarchical clustering applied only for visualization (“ggcorrplot” function from the package “ggcorrplot”). Exact mean ± SEM Spearman correlation coefficients can be found in Figure S2 of the Supplementary Material. To represent the influence of the GC all channels are visualized in the channel maps although only channels of the right hemisphere were of interest. For a better comparison the channel maps of the mean magnitudes are underlaid with the corresponding ROIs (cf. Figure 2).

**Figure 7:**
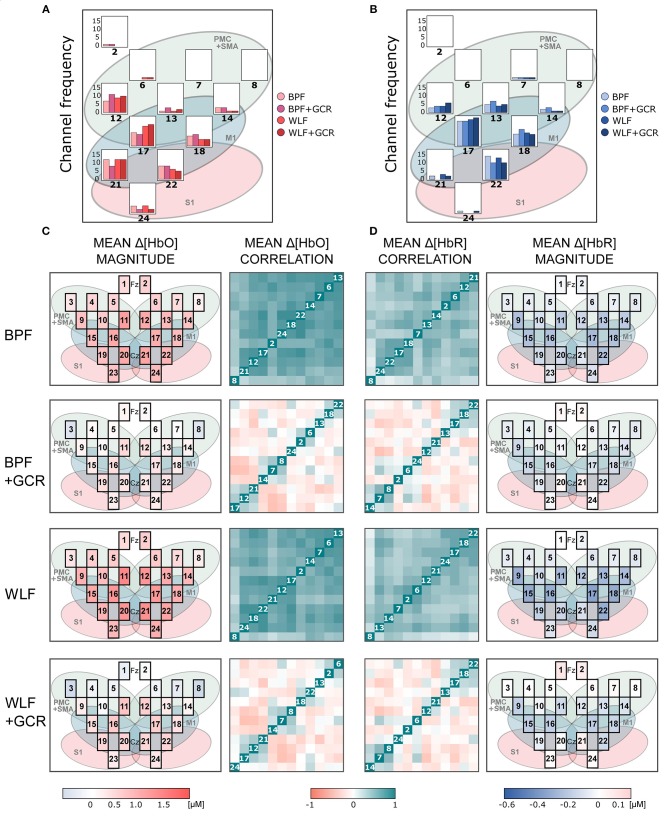
**(A,B)** Frequency of selected channels based on mean ranking of the ME and MI beta values resulting from the pre-test of **(A)** Δ[*HbO*] and **(B)** Δ[*HbR*] data. **(C,D)** Mean correlation matrices (non-normalized) and channel maps of mean magnitudes of the MI **(C)** Δ[*HbO*] and **(D)** Δ[*HbR*] data. Each row corresponds to one of the preprocessing approaches. For both signal types only WLF+GCR and BPF+GCR approaches resulted in a higher spatial specificity indicated by both mean correlation matrices and mean magnitudes. The white numbers in the diagonal correspond to the channel number. Note that the order of the channels might differ between matrices due to the hierarchical clustering applied only for visualization (“ggcorrplot” function from the package “ggcorrplot”). Exact mean ± SEM Spearman correlation coefficients can be found in Figure S2 of the Supplementary Material. To represent the influence of the GC all channels are visualized in the channel maps although only channels of the right hemisphere were of interest. For a better comparison the channel maps of the mean magnitudes are underlaid with the corresponding ROIs (cf. Figure 2).

Figures 6, 7 show the mean magnitudes and mean correlations averaged over all trials for each channel and all subjects for each preprocessing approach and separately for ME and MI as well as Δ[*HbO*] and Δ[*HbR*] data. As illustrated in Figures 6A,B, 7A,B, the channel selection is for all four preprocessing approaches comparable. However, for BPF+GCR and WLF+GCR, descriptively, on the contralateral hemisphere channels 12, 17, 18, 21, and 22 seem to capture the sensorimotor Δ[*HbO*] response best. For Δ[*HbR*], descriptively, this is the case for channels 13, 17, 18, and 22 (cf. Figures 6, 7).

The mean correlation matrices are visualized in Figures 6, 7C,D. Note that the figures show non-normalized values for a clearer comparison. Descriptively, correlation matrices are spatially unspecific and highly similar for approaches BPF and WLF, whereas for approaches BPF+GCR and WLF+GCR, although also highly comparable, correlations seem much more spatially defined. This impression was confirmed by statistical tests.

For all mean correlation matrix comparisons of MI and ME and Δ[*HbR*] and Δ[*HbO*] data, the comparison of the correlation matrices derived for the WLF and BPF as well as the comparison between BPF+GCR and WLF+GCR approaches did not result in significant differences. All other comparisons showed highly significant differences (all *p* < 0.00001; cf. Table 2). This pattern of results indicates on the one hand a strong influence of the GC on the spatial specificity of the signal and on the other hand, regarding the spatial specificity, no differences between the two applied filter types are evident. The results of all 24 correlation matrix comparisons can be found in Table 2.

**Table 2:** Spatial Specificity: Comparison of the (normalized) mean correlation matrices of Δ[*HbO*] and Δ[*HbR*] data for ME and MI Δ[*HbO*] as well as Δ[*HbR*] data.

		**Δ[*HbO*]**		**Δ**[***HbR***]****		
	**BPF**	**BPF +GCR**	**WLF**	**BPF**	**BPF +GCR**	**WLF**
**Motor execution**						
BPF +GCR	$\chi_{\left(66\right)}^{2}=$ 1379.46 *p* < 0.00001	–	–	χ^2^(66) = 664.15 *p* < 0.00001	–	–
WLF	$\chi_{\left(66\right)}^{2}=$ 1.64 *p*>0.05	$\chi_{\left(66\right)}^{2}=$ 1323.78 *p* < 0.00001	–	$\chi_{\left(66\right)}^{2}=$ 0.85 *p*>0.05	$\chi_{\left(66\right)}^{2}=$ 638.77 *p* < 0.00001	–
WLF +GCR	$\chi_{\left(66\right)}^{2}=$ 1378.46 *p* < 0.00001	$\chi_{\left(66\right)}^{2}=$ 0.42 *p*>0.05	$\chi_{\left(66\right)}^{2}=$ 1322.32 *p* < 0.00001	$\chi_{\left(66\right)}^{2}=$ 659.83 *p* < 0.00001	$\chi_{\left(66\right)}^{2}=$ 1.06 *p*>0.05	$\chi_{\left(66\right)}^{2}=$ 634.213 *p* < 0.00001
**Motor imagery**						
BPF +GCR	$\chi_{\left(66\right)}^{2}=$ 1176.21 *p* < 0.00001	–	–	χ^2^(66) = 520.89 *p* < 0.00001	–	–
WLF	$\chi_{\left(66\right)}^{2}=$ 4.67 *p*>0.05	$\chi_{\left(66\right)}^{2}=$ 1037.62 *p* < 0.00001	–	$\chi_{\left(66\right)}^{2}=$ 0.52 *p*>0.05	$\chi_{\left(66\right)}^{2}=$ 515.21 *p* < 0.00001	–
WLF +GCR	$\chi_{\left(66\right)}^{2}=$ 1179.57 *p* < 0.00001	$\chi_{\left(66\right)}^{2}=$ 0.64 *p*>0.05	$\chi_{\left(66\right)}^{2}=$ 1040.48 *p* < 0.00001	$\chi_{\left(66\right)}^{2}=$ 517.16 *p* < 0.00001	$\chi_{\left(66\right)}^{2}=$ 1.27 *p*>0.05	$\chi_{\left(66\right)}^{2}=$ 511.10 *p* < 0.00001

A second set of analyses tested for differences in the variances of single trial Spearman correlations between all contralateral channels. Regarding the ME data, the results of the Friedman tests indicated highly significant differences for the factor “approach” for both Δ[*HbO*] [$\chi_{\left(3\right)}^{2}=128.76$, *p* < 0.00001, *W* = 0.56] and Δ[*HbR*] [$\chi_{\left(3\right)}^{2}=127.32$, *p* < 0.00001, *W* = 0.48] data. The same pattern was evident for the MI data [Δ[*HbO*]: $\chi_{\left(3\right)}^{2}=131.62$, *p* < 0.00001, *W* = 0.59]; [Δ[*HbR*]: $\chi_{\left(3\right)}^{2}=132.53$, *p* < 0.00001, *W* = 0.58].

For the ME data, subsequent pairwise comparisons using Nemenyi multiple comparison test showed highly significant results (*p* < 0.05) for all comparisons except for the comparison between BPF and WLF as well as between BPF+GCR and WLF+GCR approaches. Regarding the MI data, the *post-hoc* tests showed highly significant differences (*p* < 0.01) for all comparisons besides the comparison of BPF+GCR and WLF+GCR approaches. As showed in Table 3, the variances increased to a large degree from BPF to BPF+GCR and from WLF to WLF+GCR approaches but are highly comparable between WLF and BPF and WLF+GCR and BPF+GCR. This is in support of the conclusion that removing the GC has a particulary strong influence on spatial specificity.

**Table 3:** Spatial Specificity: Statistical results from the pairwise comparison of both the motor execution and the motor imagery data as well as mean ± SEM for variances and ranks for each signal type and preprocessing approach.

**Motor execution**										
**Δ[*HbO*]**						**Δ[*HbR*]**				
$\chi_{\left(3\right)}^{2}=128.76$, *p* < 0.00001, *W* = 0.56						$\chi_{\left(3\right)}^{2}=127.32$, *p* < 0.00001, *W* = 0.48				
	**BPF**	**BPF +GCR**	**WLF**	**μ**_***rank***_ **±SEM**	**μ**_***var***_ **±SEM** [**μ*M*^2^**]	**BPF**	**BPF +GCR**	**WLF**	**μ**_***rank***_ **±SEM**	**μ**_***var***_ **±SEM** [**μ*M*^2^**]
BPF	–	–	–	47.22 ±3.97	83.82 ±8.61	–	–	–	47.14 ±4.03	184.87 ±10.76
BPF +GCR	*p* < 0.00001	–	–	153.26 ±4.21	453.39 ±6.27	*p* < 0.00001	–	–	152.52 ±4.36	465.55 ±5.72
WLF	*p*>0.05	*p* <0.00001	–	53.78 ±4.21	98.04 ±9.53	*p*>0.05	*p* < 0.00001	–	54.78 ±4.39	208.01 ±0.06
WLF +GCR	*p* < 0.00001	*p*>0.05	*p* < 0.00001	147.74 ±3.99	444.58 ±5.58	*p* < 0.00001	*p*>0.05	*p* < 0.00001	147.56 ±4.04	460.63 ±5.21
**Motor imagery**										
**Δ[*HbO*]**						**Δ[*HbR*]**				
$\chi_{\left(3\right)}^{2}=131.62$, *p* < 0.00001, *W* = 0.59						$\chi_{\left(3\right)}^{2}=132.53$, *p* < 0.00001, *W* = 0.58				
	**BPF**	**BPF +GCR**	**WLF**	**μ**_***rank***_ **±SEM**	**μ**_***res***_ **±SEM** [**μ*M***^2^]	**BPF**	**BPF +GCR**	**WLF**	**μ**_***rank***_ **±SEM**	**μ**_***res***_ **±SEM** [**μ*M*^2^**]
BPF	–	–	–	44.84 ±4.14	97.12 ±7.99	–	–	–	45.38 ±4.00	179.05 ±9.29
BPF +GCR	*p* < 0.00001	–	–	150.44 ±4.19	413.41 ±4.74	*p* < 0.00001	–	–	146.20 ±4.11	428.51 ±5.60
WLF	*p*<0.01	*p* < 0.00001	–	56.16 ±3.95	117.80 ±8.23	*p*<0.01	*p* < 0.00001	–	55.66 ±4.12	205.34 ±9.86
WLF +GCR	*p* < 0.00001	*p*>0.05	*p* < 0.00001	150.56 ±4.05	412.60 ±4.46	*p* < 0.00001	*p*>0.05	*p* < 0.00001	154.76 ±4.05	441.00 ±6.03

### 4.3. Consequences for Statistical Outcomes

#### 4.3.1. Motor Execution: Time on Task Comparison

In order to compare the approaches with respect to experimental outcomes 4 ×2 ANOVAs with repeated measures and the within-subject factors “approach” (BPF, WLF, BPF+GCR, WLF+GCR) and “time on task” (pre, post) were conducted.

The results regarding the Δ[*HbO*] data indicated a highly significant main effect of “time on task” [*F*_(1, 49)_ = 9.03, *p* < 0.01, $\eta_{G}^{2}=0.03$] with a decreasing mean value from pre-test (3.53±0.18μ*M*) to post-test (2.91±0.15μ*M*). Furthermore, the main effect of “approach” [*F*_(3, 147)_ = 139.08, *p* < 0.00001, $\eta_{G}^{2}=0.34$] as well as the interaction “approach:time on task” [*F*_(3, 147)_ = 7.84, *p* < 0.001, $\eta_{G}^{2}=0.006$] were highly significant.

The 4 × 2 ANOVA for Δ[*HbR*] indicated only a highly significant main effect for “approach” [*F*_(3, 147)_ = 83.54, *p* < 0.00001, $\eta_{G}^{2}=0.20$]. Regarding the main effect of “approach,” the pairwise comparisons are documented in Tables 4, 5 and showed for all comparisons a highly significant difference (*p* < 0.00001) for Δ[*HbO*] and Δ[*HbR*] data, respectively.

**Table 4:**
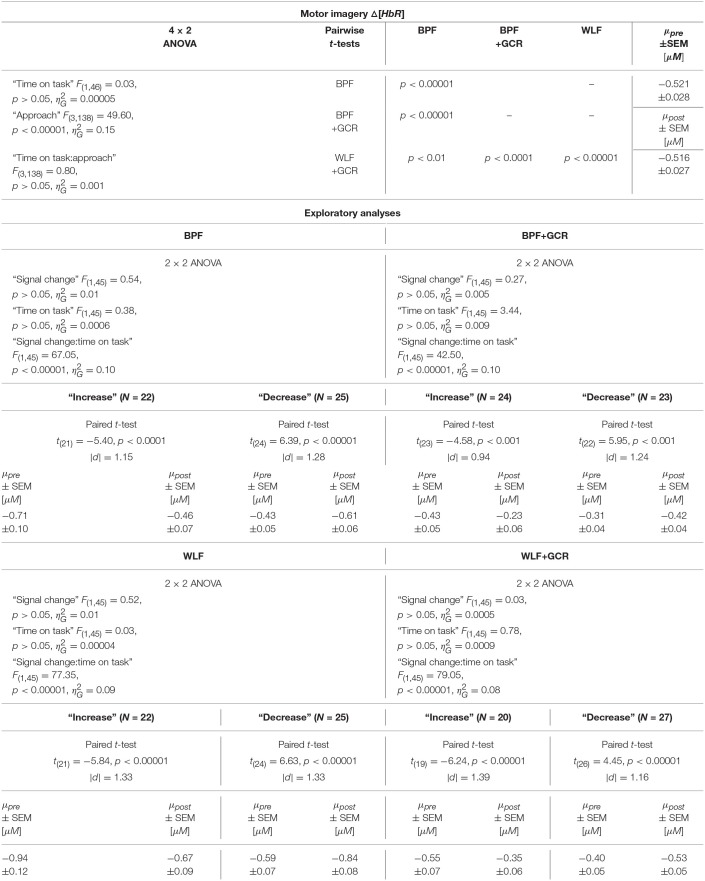
Consequences for Statistical Outcome: Results of all statistical tests applied regarding the ME Δ*HbO* data, including main tests, *post-hoc* tests and exploratory analyses.

**Table 5:**
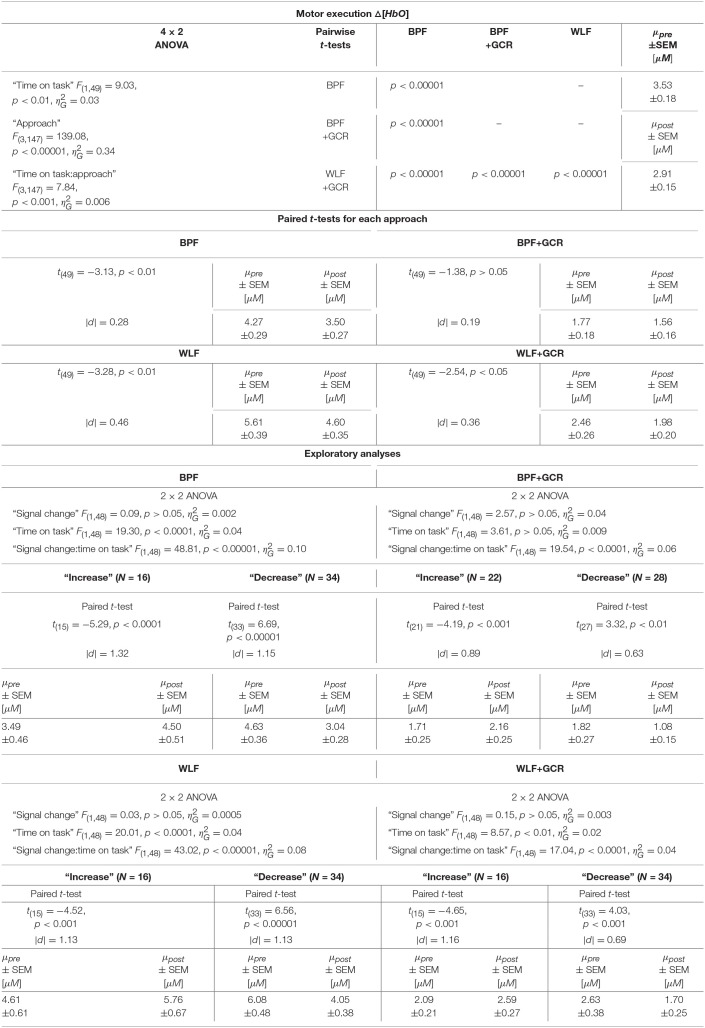
Consequences for Statistical Outcome: Results of all statistical tests applied regarding the ME Δ*HbR* data, including main tests, *post-hoc* tests and exploratory analyses.

**Figure 8:**
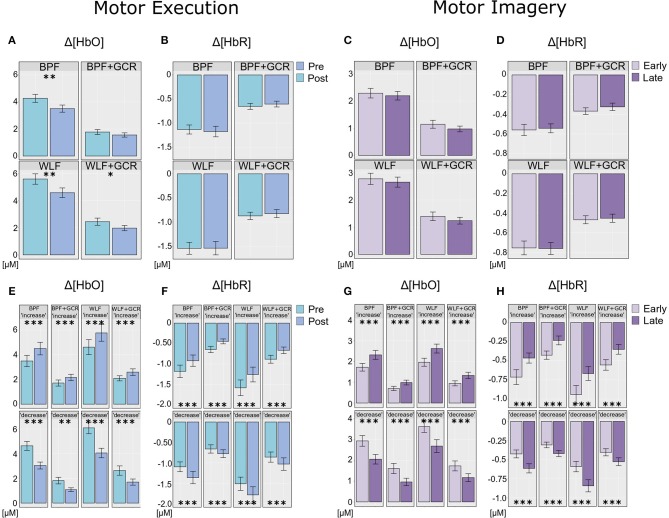
**(A,B)** Differences of **(A)** Δ[*HbO*] and **(B)** Δ[*HbR*] ME data between the preprocessing approaches from pre- (bright blue) to post-test (dark blue). **(E,F)** ME data after exploratory regrouping of data in “increase” and “decrease” signal change groups for each approach, **(E)** for Δ[*HbO*] and **(F)** for Δ[*HbR*] data. **(C,D)** Differences of **(C)** Δ[*HbO*] and **(D)** Δ[*HbR*] MI data between the preprocessing approaches from early (bright violet) to late MI (dark violet). **(G,H)** MI data after exploratory regrouping of data in “increase” and “decrease” signal change groups for each approach, **(G)** for Δ[*HbO*] and **(H)** for Δ[*HbR*] data. Error bars show standard error of the mean. Significance level: *p < 0.05, **p < 0.01, ***p < 0.001.

To further explore the interaction for each preprocessing approach a paired *t-*test was conducted with Δ[*HbO*] as response variable and “time on task” (pre, post) as explanatory variable. Except for the BPF+GCR approach all t-tests showed significant differences with a general direction of a decrease from pre- to post-test (see Figure 8A). The size of the statistical effects decreased from WLF approach (|*d*| = 0.46) to the WLF+GCR approach (|*d*| = 0.36) to the BPF method (|*d*| = 0.28) to the to the BPF+GCR approach (|*d*| = 0.19).

#### 4.3.2. Motor Imagery: Time on Task Comparison

For the MI data, 4 × 2 ANOVAs with the within-subject factors “approach” (BPF, WLF, BPF+GCR, WLF+GCR) and “time on task” (early, late) were performed to assess the effect of preprocessing approach on experimental effects.

For both the Δ[*HbO*] and the Δ[*HbR*] data, a highly significant main effect of “approach” was found [Δ[*HbO*]: *F*_(3, 138)_ = 101.19, *p* < 0.00001, $\eta_{G}^{2}=0.28$; Δ[*HbR*]: *F*_(3, 138)_ = 49.60, *p* < 0.00001, $\eta_{G}^{2}=0.15$]. For the Δ[*HbO*] data, the pairwise comparisons indicated a highly significant difference between all approaches (*p* < 0.00001) except for the comparison of the BPF and WLF methods (*p*>0.05).

Neither the main effect of “time on task” nor an interaction between the two factors was significant for both signal types. This is illustrated in Figures 8C,D. Regarding both the Δ[*HbO*] and the Δ[*HbR*] data, all pairwise comparisons resulted in significant differences (*p* < 0.05).

#### 4.3.3. Additional Exploratory Analyses

As evident from the statistical analyses and as illustrated in Figures 8B–D, “time on task” had no effect on the ME Δ[*HbR*] data as well as on the MI Δ[*HbO*] and Δ[*HbR*] data signal irrespective of preprocessing approach. Exploratory analyses were conducted to better understand this unexpected result. For the sake of completeness, although a main effect of “time on task” was evident for ME Δ[*HbO*] data, the exploratory analyses were also applied for this data.

A possible explanation of the lacking “time on task” effect could be that part of the subjects showed a decrease in signal over time, while the remaining subjects showed an increase, effectively averaging out any “time on task” effect at the group level. To test this idea, the sample was regrouped into a group with a signal increase (“increase”) and a signal decrease group (“decrease”), separately for ME and MI, each preprocessing approach and for Δ[*HbO*] and Δ[*HbR*] data. This grouping was based on the difference of the mean signal value of pre- and post-test for ME and early and late data for MI, respectively. For ME and MI, each approach and signal type (Δ[*HbO*] and Δ[*HbR*]) a 2 × 2 mixed repeated measures ANOVA with within-subject factor “time on task” [pre, post (ME) or early, late (MI)] and between-subjects factor “signal change” (increase, decrease) was conducted.

All test results, effect sizes and group sizes are given in Tables 4–7.

##### 4.3.3.1. Motor Execution

Regarding the ME data, the output of the ANOVAs are documented in Tables 4, 5. Overall, the effect of most interest was the interaction “signal change:time on task” which was for both Δ[*HbO*] and Δ[*HbR*] data and all preprocessing approaches highly significant (*p* < 0.00001; cf. Tables 4, 5). To further explore this interaction, paired *t*-tests were conducted for both Δ[*HbO*] and Δ[*HbR*] data and each preprocessing approach separately in order to find a potential “time on task” effect for the signal change subgroups. As illustrated in Figures 8E,F, the results of all applied *t*-tests showed highly significant differences between pre- and post-test data (*p* < 0.001).

For the Δ[*HbO*] data and all preprocessing approaches, the “decrease” signal change group comprised the larger amount of subjects as compared to the “increase” signal change group (Δ*N* = 6 for BPF+GCR and Δ*N* = 18 for all other approaches) which might be the reason for the main effect of “time on task” of the primary analysis (cf. section Motor Execution: Time on Task Comparison), which resulted in a signal decrease from pre- to post-test. For the Δ[*HbR*] data the size of the subgroups are more comparable (Δ*N*: 2–8) and is likely to be the reason for the lacking “time on task” effect in the main analysis (cf. section Motor Execution: Time on Task Comparison).

For all subgroups of both Δ[*HbO*] and Δ[*HbR*] data and each preprocessing approach the effect sizes always decreased from BPF to BPF+GCR and from WLF to WLF+GCR.

##### 4.3.3.2. Motor imagery

In terms of the MI data, neither a significant main effect of “signal change” nor of “time on task” was evident. However, highly significant interactions between the two factors were found for all four preprocessing approaches (*p* < 0.0001; cf. Tables 6, 7).

**Table 6:**
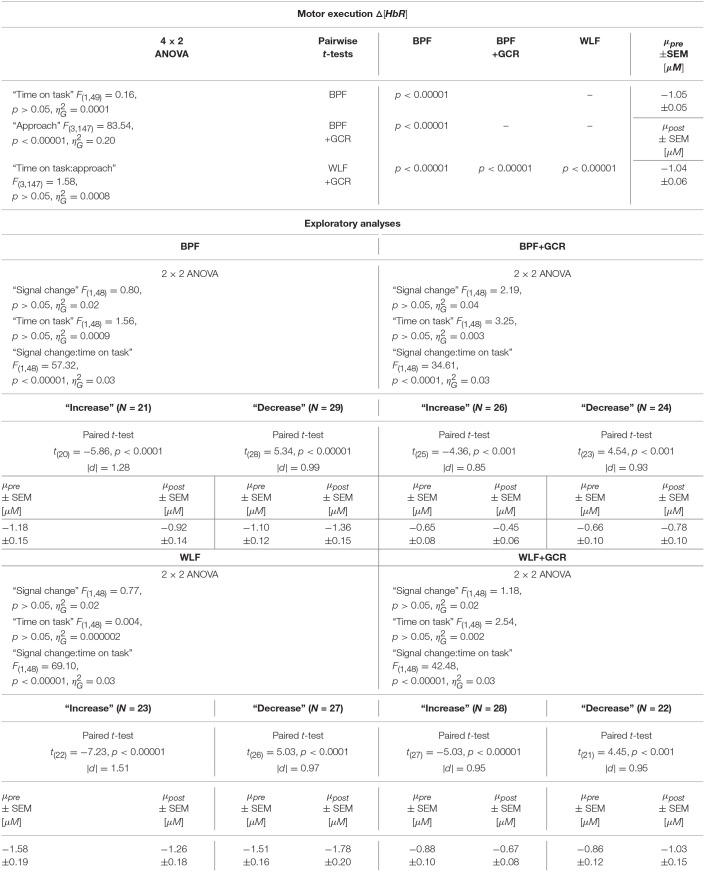
Consequences for Statistical Outcome: Results of all statistical tests applied regarding the MI Δ*HbO* data, including main tests, *post-hoc* tests and exploratory analyses.

**Table 7:**
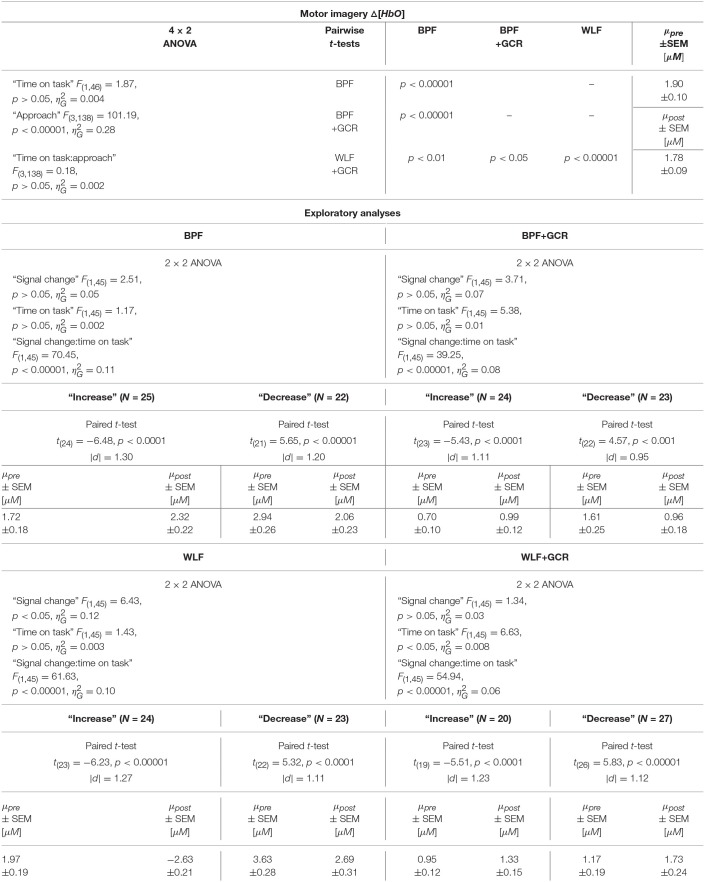
Consequences for Statistical Outcome: Results of all statistical tests applied regarding the MI Δ*HbR* data, including main tests, *post-hoc* tests and exploratory analyses.

In order to explore the significant interactions, paired t-tests were performed to test for differences between early and late MI data in the respective subgroups. The results are summarized in Tables 6, 7 and visualized in Figures 8G,H. All comparisons showed a highly significant difference between early and late MI trials (*p* < 0.001).

As for the ME Δ[*HbR*] data, for both the MI Δ[*HbO*] and Δ[*HbR*] data and all preprocessing approaches, the size of the subgroups are comparable (Δ*N*: 1–7) and might be also here the reason for the lacking main effect in the primary analysis (cf. section Motor Imagery: Time on Task Comparison).

Regarding the effect sizes, for both Δ[*HbO*] and Δ[*HbR*] data the effect sizes decreased in both subgroups from BPF to BPF+GCR approaches. For both subgroups of the Δ[*HbR*] data there was also a decrease in effect size from WLF to WLF+GCR approaches. However, for the Δ[*HbO*] data only the “increase” subgroup showed a decrease in effect size from WLF to WLF+GCR whereas the effect size of the “decrease” group minimally increased.

Taken together, the results of the exploratory analyses indicate that even though no experimental effect is evident for both the Δ[*HbO*] and Δ[*HbR*] data of the MI training phase as well as the Δ[*HbR*] data of the ME tests when considering the whole sample, significant experimental effects are evident for “signal change” subgroups. Because these experimental effects are of opposing direction they are annulled in the sample average. However, no clear pattern emerged with regard to the relationship between preprocessing approach and size of the “time on task” effect but, except for the comparison of the “decrease” group of the ME Δ[*HbO*] data, a decrease of effect size from one approach (BPF or WLF) to its more elaborate version (BPF+GCR or WLF+GCR) is evident. Notably, subgroup assignment was influenced by preprocessing approach. That is, at least for some participants the preprocessing approach determined whether Δ[*HbO*]/Δ[*HbR*] magnitudes increased or decreased as a function of “time on task.”

## 5. Discussion

The present study investigated the effects of four fNIRS preprocessing approaches on single trial data by means of temporal consistency between Δ[*HbO*] and Δ[*HbR*], and of spatial specificity of either signal. In addition, experimental outcomes were compared between preprocessing approaches. The preprocessing approaches chosen were conventional band-pass filtering (BPF), a wavelet MDL detrending filter (WLF; Jang et al., 2009) and the combination of each approach with global component removal (WLF+GCR; Zhang et al., 2016 and BPF+GCR). It was expected that the more elaborate approaches lead to a higher signal quality and consequently in a higher temporal consistency between Δ[*HbO*] and Δ[*HbR*] and a greater spatial specificity of the signals. Experimental effects were expected to be reduced for the more elaborate preprocessing approaches as compared to BPF and WLF alone.

### 5.1. Temporal Consistency

Descriptively, and as illustrated in Figures 4, 5, against the expectations, for the WLF approach, in most cases signal amplitude and morphology were quite similar to that resulting from the BPF approach. However, as hypothesized after the combination of each filtering method with the GCR method, the signal amplitude was greatly reduced as compared to the BPF and WLF alone (cf. Figures 4, 5). Furthermore, multiple peaks were strongly suppressed, suggesting an origin in evoked systemic activity. Temporal consistency was statistically assessed based on the residuals of the linear models fitted for Δ[*HbO*] and Δ[*HbR*] data, where a small residual would indicate clean data and high signal quality (Zhang et al., 2016).

It was hypothesized that the WLF method results in a higher temporal consistency as compared to the BPF method, consequently a further increase in temporal consistency was expected from BPF+GCR to WLF+GCR. This was not confirmed by means of the statistical results. Although, for both the ME and the MI data, the mean residuals resulting from preprocessing with the BPF and WLF approach were nearly twice as high as those resulting from the BPF+GCR and WLF+GCR approaches (cf. Table 1), the temporal consistency decreased from BPF to WLF and from BPF+GCR to WLF+GCR. Overall and independent of whether the channel selection was based on BPF+GCR or WLF+GCR, the lowest residuals resulted from the BPF+GCR approach which was also confirmed by statistical outcomes.

For block averaged data and using the WLF+GCR approach only, Zhang et al. (2016) proposed that a decrease in residuals reflects higher data quality. The results of the present study indicate that both BPF+GCR and WLF+GCR strongly reduce residuals also when applied to single trial data, and, following the notion of reduced residuals reflecting higher data quality, specifically BPF+GCR can be assumed to significantly improve single trial signal quality.

### 5.2. Spatial Specificity

Spatial specificity was quantified by means of (contralateral) channel correlation matrices and (contralateral) channel variance comparisons. Mean normalized correlation matrices differed significantly between all preprocessing approaches of both MI and ME and Δ[*HbO*] and Δ[*HbR*] data except for the comparison of BPF and WLF and of BPF+GCR and WLF+GCR approaches. Whereas for the BPF and WLF approaches nearly all contralateral channels were highly positively correlated in spite of considerable distances between the channels, descriptively, for the BPF+GCR and WLF+GCR approaches only adjacent channels formed positive correlation clusters, and negative and zero-correlation clusters appeared to be present as well (cf. Figures 6, 7). Variance increased significantly from BPF to BPF+GCR and from WLF to WLG+GCR, indicating that channel signals became increasingly independent from one another. However, likewise in these analyses no difference between BPF and WLF and between BPF+GCR and WLF+GCR approaches for the ME data and between BPF and WLF approaches in the MI data were evident. These results were unexpected in that it was hypothesized that WLF resulted in higher spatial specificity as compared to BPF, followed by BPF+GCR to WLF+GCR.

Taken together, differences in correlation matrices and the increase in variance support the conclusion that the removal of the global component results in a more specific signal. Furthermore, these results indicate no difference in terms of spatial specificity between BPF and WLF approaches.

The increased spatial specificity in particular for the BPF+GCR and WLF+GCR preprocessed data was clearly evident in the mean activation maps (cf. Figures 6, 7). Here, for Δ[*HbO*] and Δ[*HbR*] the contralateral channels with the largest signals (respectively 12, 17, 21, and 22; and 13, 17, 18, and 22) covered supplementary motor and premotor areas (SMA/PMC) and primary motor areas (M1). This is in line with typical activation patterns for sequential tasks found in MI and motor learning research (Dayan and Cohen, 2011; Mizuguchi and Kanosue, 2017).

Interestingly, a high influence of the GC on spatial specificity was evident for both Δ[*HbO*] and Δ[*HbR*] data. This is in contrast to previous findings (Yücel et al., 2015; Zhang et al., 2016) where it has been suggested that mostly Δ[*HbO*] data are affected by global component activity. At present, the cause of this discrepancy is not clear, but our results should caution against relying on uncorrected Δ[*HbR*] data in the hope that here the GC poses no problem.

### 5.3. Consequences for Experimental Outcomes

For both ME and MI and respectively Δ[*HbO*] and Δ[*HbR*], the data resulting from the four preprocessing approaches differed highly significantly from each other. In addition, regarding the ME data, for the Δ[*HbO*] data a significant “time on task” effect was evident as well as an significant interaction between the effects of “preprocessing approach” and “time on task.” The largest effect size for “time on task” was found for the WLF approach, followed by the WLF+GCR and then the BPF and BPF+GCR approaches. Concerning the MI data, the experimental factor “time on task” was neither associated with a significant main effect nor a significant interaction. Subsequent exploratory analyses performed on “signal change”-based subsamples indicated that “time on task” effects were present and that they varied between preprocessing approaches. However, the expected decrease in effect size of the “time on task” effect from BPF to BPF+GCR and from WLF to WLF+GCR could not be reliably observed across all subsamples.

The pattern observed for ME data indicates that for an experimental task where a strong and reliable hemodynamic response can be expected, experimental outcomes will likely be more affected by artifactual sources when based on Δ[*HbO*] as compared to Δ[*HbR*] data, as reported in previous studies (Zhang et al., 2016; Pfeifer et al., 2018). Yet when responses are smaller and perhaps less reliable as for MI the present results suggest that this might not hold true, as experimental effects varied with preprocessing approach both for Δ[*HbO*] and Δ[*HbR*]. This variation indicates that the presumed insensitivity of Δ[*HbR*] to artifacts might be a misconception. However, MI results were derived from exploratory analyses and rely on a much smaller sample than those obtained for ME, hence future research is needed to consolidate this observation.

### 5.4. Constraints and Conclusions

The results of the present study are object to a number of constraints. Regarding the GCR approach (Zhang et al., 2016) a possible issue is the optode coverage, which the authors of the approach recommended to be “much larger than the expected pattern of activity” (Zhang et al., 2016) due to the large-sized kernel. More specifically, they indicate the coverage to be 9 cm or more. Though total coverage in the present study was not as extensive as in Zhang et al. (2016), the subset of channels selected for GCR and hence relative coverage was comparable between the studies and fit the 9 cm criterion. As visualized in Figures 6C,D, 7C,D, applying the GC removal results for some of the frontal channels in a reversed hemodynamic response. Reversed response patterns have been reported before for motor imagery tasks (Holper et al., 2011; Kober and Wood, 2014), and were for instance interpreted as a sign of motor inhibition due to a reduction of activation of the underlying brain regions (Kober and Wood, 2014). Alternatively, these negative responses could also be due to the fact that the SVD algorithm is not able to account for local differences within the global physiological changes. Hence, it offers a good but not perfect estimation of the systemic activity and therefore the results should always be considered with care.

GCR, in combination with either WLF and BPF, might be an option not only for offline analyses of averaged and single trial data, but also for fNIRS online implementations such as intermittent neurofeedback. Intermittent neurofeedback describes the situation where, in contrast to continuous neurofeedback, feedback on brain responses is given only following the end of a trials' task period, giving room for some online signal correction. Besides, regarding nowadays' computer hardware, also the implementation of these approaches in a continuous online experiment should be possible since these approaches are not computationally demanding. Before being used for this purpose, the approach should however be validated online, in line with a recent proposal regarding the validation of methods and approaches intended for EEG-based brain-computer interfaces (Lotte et al., 2018).

In single-trial analyses in general and in neurofeedback or BCI setups specifically, clean signals are of particular relevance to ensure reliable measurements and, for neurofeedback, to ensure that a functionally specific modulation of brain activity can be achieved. The results of the present study show that for ME and MI both the WLF+GCR and the BPF+GCR are promising preprocessing approaches to obtain cleaner signals, as it was consistently linked to single trial signal improvement in terms of temporal consistency and spatial specificity. More generally, this studies' results highlight the tremendous need for an agreement on appropriate preprocessing pipelines in fNIRS. Notably, for other tasks which might not result in a strong hemodynamic response, as is typically found in motor tasks, a proper correction is not guaranteed.

Notwithstanding, the major constraint in the present study is the lack of a validation by means of a short-distance channel approach, that would allow to measure and remove the extracerebral component of the GC, and similarly, a means of validation the removal of the cerebral component of the GC. Thus, our results, similar to those of others (Zhang et al., 2016; Pfeifer et al., 2018), should be considered tentative until corresponding data sets and analyses are available.

## Data Availability Statement

The datasets generated for this study are available on request to the corresponding author.

## Ethics Statement

The study was approved by the Ethics Committee of the University of Oldenburg.

## Author Contributions

FK and CK designed the study. FK collected and analyzed the data as well as wrote the first draft of the manuscript. CK supervised FK and edited as well as commented the manuscript in all of its versions.

### Conflict of Interest

The authors declare that the research was conducted in the absence of any commercial or financial relationships that could be construed as a potential conflict of interest.
