# Supplementary material for: Signal Processing in fNIRS: A Case for the Removal of Systemic Activity for Single Trial Data
Source: Front Hum Neurosci. 2019 Sep 24;13:331. doi: 10.3389/fnhum.2019.00331 (PMC6769087; doi:10.3389/fnhum.2019.00331)
Supplement: Supplementary file 1 [file Data_Sheet_1.PDF]

Supplementary Material

1 GROUP COMPARISONS

The data analysed in this paper was originally collected in the context of a fNIRS-based MI neurofeedback group comparison. In the MI training phase two groups received intermittent feedback based on their $\triangle[HbO]$ signal (N = 20 in total, N = 17 entered analysis) and $\triangle[HbR]$ (N = 20 in total, N = 16 entered analysis) signal, respectively. A control group performed the same MI task without receiving any feedback (N = 20 in total, N = 17 entered analysis). Since no group differences were found, neither in the signal nor bahviorally, the subjects of all three groups were merged into a single data set for the analysis of the present study.

2 EXPERIMENTAL DESIGN

Each subject participated in one experimental session consisting of familiarization phase, pre-test (ME and MI), training session (MI) and post-test (ME). During the session, subjects sat comfortably in a chair with their arms on the armrests in a sound attenuated, dimly lit booth. Participants sat in front of a computer monitor at a distance of approximately 175 cm. The motor task was a self-paced sequential 8-position finger-tapping task. Participants were instructed to use the left hand for the task and that for the motor task each finger corresponds to a specific number (i.e., index finger = 2, middle finger = 3, ring finger = 4 and pinky finger = 5). During the whole experiment the participants were instructed to place their left hand on a number pad and to not move their right hand. The right hand rested on the participant's lap. In the familiarization phase and the pre- and post-test session Presentation software (Version 18.01 Build 01.04.16, Neurobehavioral Systems, Inc., Berkeley, CA, www.neurobs.com) was used to present the visual stimuli, in the training session the stimuli were presented by means of the Psychophysics toolbox (Version 3;Brainard, 1997; Pelli, 1997; Kleiner et al., 2007) in MATLAB 2017a (The MathWorks Inc., Natick, MA, USA) to make it compatible with the toolbox Turbo-Satori (v0.8) used for feedback generation (Lührs and Goebel, 2017).

2.1 Familiarization Phase, Pre- and Post-Test

At the beginning of the experiment participants were asked to memorize a sequence of numbers (3-2-3-5-4-2-5-4). Before memorizing the sequence, they were reminded that each number corresponded to a specific finger. Participants were allowed to project the numbers to their left hand fingers, but were not allowed to move their fingers while learning the sequence. Subjects had no time pressure whilst learning the sequence and let the experimenter know when they were able to start. After this memorization phase they were asked to type the sequence on the number pad. Every time a sequence was completed, subjects were instructed to indicate this by pressing the enter button. They also had the opportunity to press this button when they recognized a mistake while typing in the sequence and to start again with a new sequence. In this phase the participants had 10 attempts to correctly type in the sequence and were told that they would progress to the next stage of the experiment when they typed in five consecutive sequences correctly. All 50 subjects of the final sample achieved this criterion.

In the first part of the pre-test and for the post-test the subjects were instructed to physically perform the task with their left hand by typing in the sequence learned in the familiarization phase and to indicate the end of each sequence by pressing the enter button with the thumb of the left hand. This had to be repeated for the 20s execution task period indicated by a blue fixation cross displayed on the computer screen. Each task period was preceded by a pseudo-randomized rest period lasting from 18 to 22s indicated by a red

fixation cross. For the rest period subjects were instructed not to move their hands at all and only to fixate the cross on the screen followed after this task period. Each rest and each task period was preceded by a 1s dark red fixation cross to indicate to the participats a change between the periods. All subjects performed five ME trials (task + rest period) and were instructed to type in as many correct sequences as fast as possible during the task period. Subjects were also encouraged to try to increase the number of sequences from trial to trial. As in the familiarization phase, subjects could abort a sequence when realizing they had made a mistake by pressing the enter button before continuing with a new sequence. The total duration of the five ME trials never exceeded 220s.

For the pre-test, the ME phase owas followed by an MI phase in which subjects mentally performed the finger-tapping task with their left hand. All instructions were essentially the same as for ME but instead of physically performing the finger-tapping task the participants had to kinesthetically imagine to type in as many correct sequences as possible and as fast as possible for 20s per trial. Subjects were asked not to move their hands at all times. Participants performed five MI trials that lasted no longer than 220 sec in total. The complete pre-test lasted no longer than 10 minutes. The post-test consisted only of ME trials and lasted no longer than 5 minutes, including the instructions and questions on the part of the subjects.

2.2 Online Training Session

In the training session subjects were instructed to mentally perform the motor task by kinesthetically imagining to type in as many correct sequences as possible and as fast as possible and to finish each sequence by imagining to press the enter button. The training session consisted of 20 MI trials. Task period of a trial lasted again 20s and was indicated by a blue fixation cross on the computer screen. It was preceded by a rest phase lasting for 18-22s, for which the participants were instructed not to move their hands and to fixate the red cross on the screen. In contrast to the MI phase of the pre-test, each MI task phase was followed by a moving thermometer bar. The meaning of this bar differed between groups.

In the feedback groups participants received visual feedback based on their MI-based signal, that is, either on $\triangle[HbO]$ or $\triangle[HbR]$. Visual feedback lasted for 4s and was given following the task period by means of a moving thermomemter bar that filled in accordance to the feedback value calculated online. Details on feedback calculation are given in section 2.4. Participants belonging to these groups were informed about the meaning of the feedback bar and were instructed to attempt to increase the bar from trial to trial by trying to intensify the imagination of the motor task.

For the control group the moving thermometer bar remained on the computer screen for 4s and always filled up from bottom (0) to top (100). The explanation provided to the subjects was that the bar displays the time for the next rest period to start. The time bar was included to make the setup for the control condition visually as close as possible to the setup of the feedback groups. For all three groups, the training session of all three groups consisted of 20 trials and lasted no longer than 25 minutes including time for technical adjustments (e.g., channel selection), instructions and answering questions. In total, the whole session (familiarization phase, pre-test, training and post-test) lasted no longer than 45 minutes.

2.3 Channel Selection and Start Value

Before MI training started a localizer script was applied to select the individual feedback channel. In a first step, the concentration changes recorded in the pre-test ME and MI pre-test trials were calculated using the mBLL with the nilab2 toolbox (Rev. 2.0, NIRx Medizintechnik GmbH, Berlin, Germany) in MATLAB 2012a. Thereupon, the data was band-pass filtered ($0.01 - 0.7$ Hz) and a GLM was run. Afterwards, the data was detrended, epoched (-10 to 20 sec around stimulus onset) and baseline corrected (-10 to -1 sec). In a second step, based on the beta values derived from the GLM, all contralateral channels were ranked once for ME and once for MI data. The ranks for ME and MI were then averaged for every channel and a

second ranking was performed for the average ranks. The channel with the highest rank was then chosen as feedback channel. In case two or more channels had the same rank and additional criterion was ntroduced to choose from these channels. This additional criterion was the product of the mean concentration change of ME trials and of MI trials for a window of $\pm$ 2s around the highest peak. The channel with the highest product was chosen for feedback.

For the provision of feedback, in addition, a start value for the thermometer bar was required. The start value corresponded to the individual 100% mark of the visual feedback for the first MI neurofeedback trial. The value was derived from the five MI trials of the pre-test. From these trials the trial with the highest peak in a window of 4 to 18s after onset of the MI task was chosen. This window was selected because the hemodynamic response is not expected to peak before 4-6s after task onset due to the delayed nature of the signal. The mean over a window of $\pm$ 2s around this peak was calculated. For the start value, this value was increased by 20% and set as the 100% mark of the feedback bar.

2.4 Feedback Protocol

For online feedback, data was preprocessed with Turbo-Satori (v0.8), a toolbox for real-time fNIRS neurofeedback and brain-computer interfacing Lührs and Goebel (2017). This toolbox communicates directly with the NIRx software (NIRStar 14.2; NIRx Medizintechnik GmbH, Berlin, Germany) and allows the combination with a third party stimulus application software. Turbo-Satori then sent the real-time preprocessed hemoglobin data into MATLAB where the visual feedback was generated. The online preprocessing included the conversion of the raw fNIRS signal into the hemoglobin concentration changes via the mBLL and band-pass filtering using a first order butterworth low-pass filter with a cut-off frequency of 0.7 Hz and a third order butterworth high-filter with a cut-off frequency of 0.01 Hz. The preprocessed data was then directly transmitted to MATLAB 2017a (The MathWorks Inc., Natick, MA, USA) via a network interface offered by Turbo-Satori. The hemoglobin data of the last 10s of the rest period (baseline; BL) and for the whole 20s of the MI task period was accessed. The data was detrended and the BL data was additionally averaged to baseline-correct the MI data.

Since intermittent feedback was generated, the feedback value was calculated after a trial (rest + MI period) was completed. Therefore, the maximum value of either $\triangle[HbO]$ or $\triangle[HbR]$ in a window ranging from 4 to 18s after stimulus onset was selected, the mean value over the $\pm$ 2s window around this value was calculated and the averaged BL data was subtracted. The feedback value for a given trial was then computed as the ratio of the start value and this mean value (in percent). The visual feedback was presented after the MI phase as a moving thermometer bar that started empty (0%) and then filled up till the feedback value for the current trial was reached. The feedback bar could not exceed 100% or undershoot 0% even if the feedback value was higher than 100% or lower than 0%, respectively.

3 ELECTROMYOGRAPHY (EMG)

EMG data was collected in order to control for any voluntary movements during the MI trials.To identify MI trials with movement, a classifier was trained on independent training data to distinguish movement and no movement trials (Classification Learner App; The MathWorks Inc., Natick, MA, USA). The training set comprised left hand EMG data of movement and no movement segments recorded from a single subject in three different sessions conducted at three different days. The electrode placement was identical to that used in the main experiment (cf. Figure S2). The general preprocessing was identical for both the training and the test data sets and was done by means of the EEGLAB toolbox (v14.1.1b; Delorme and Makeig, 2004) using MATLAB R2017b (The MathWorks Inc., Natick, MA, USA). The imported EMG data was high-pass filtered with a cut-off frequency of 20 Hz and a filter order of 660 in order to keep only

the muscle activity. A wavelet denoising approach was applied (Wavelet Signal Denoiser toolbox; The MathWorks Inc., Natick, MA, USA) with a Daubechies 4 (db4) wavelet. After epoching the data, a root mean square envelope of the rectified signal for each epoch was calculated and the epochs were cut into 9s segments.

The training data comprised 318 epochs (0 to 18s after stimulus onset), including 158 no movement and 160 movement trials. After preprocessing, a total number of 636 9s segments (316 no movement and 320 movement) was available to train the classifier. The feature selection for both the training set and for the data to be classified comprised variance, mean, standard deviation, log detector, wavelength and the maximum value of each of these 9 sec segments (Phinyomark et al., 2012). The selected classifier was an ensemble bagged trees algorithm with an accuracy of 96.7%. Ensemble methods use several decision trees resulting in a better precision as compared to a single decision tree. Bagging or bootstrap aggregation is applied to reduce the variance of the decision trees. The MATLAB-based app (Classification Learner App; The MathWorks Inc., Natick, MA, USA) has Breiman's 'random forest' algorithm implemented (Breiman, 2001). For this algorithm, several randomly chosen subsets are generated from the training set. By means of this collection of subsets the decision trees are trained (ensemble), resulting in a more robust average of all predictions of the different trees which is used to classify the data (Dietterich, 2000).

The epochs of the test data (each subject separately) included the ME data of the left hand and the MI data of both hands. Epochs covered -10 to 17s around stimulus onset and were cut into three 9s segments. The same features as used for the training were extracted to classify the trials with respect to movement and no movement. It was determined that a participant would be excluded from the data set when either one or more of the left hand ME trials (real movement) were not correctly classified as movement trials or more than 15 MI trials of the feedback session had to be excluded due to movement (including both left and right hand). An MI trial was identified as a 'movement trial' when one or more of its three 9s segments were classified as movement, regardless of hand. This resulted in the exclusion of ten participants. Three subjects failed the first criterion (incorrect classification of ME trials) and seven did not meet the second criterion (less than five MI trials remained for analysis), resulting in a total of 50 subjects remaining for analysis.

REFERENCES

Brainard DH. The psychophysics toolbox. *Spatial Vision* **10** (1997) 433–436.

Pelli DG. The videotoolbox software for visual psychophysics: Transforming numbers into movies. *Spatial Vision* **10** (1997) 437–442.

Kleiner M, Brainard DH, Pelli D, Ingling A, Murray R, Broussard C. "what's new in psychtoolbox-3?". *Perception* **36** (2007) 1–16.

Lührs M, Goebel R. Turbo-satori: a neurofeedback and brain-computer interface toolbox for real-time functional near-infrared spectroscopy. *Neurophotonics* **4** (2017) 041504. doi:10.1117/1.NPh.4.4.041504.

Delorme A, Makeig S. Eeglab: an open source toolbox for analysis of single-trial eeg dynamics including independent component analysis. *Journal of neuroscience methods* **134** (2004) 9–21. doi:10.1016/j.jneumeth.2003.10.009.

Phinyomark A, Phukpattaranont P, Limsakul C. Feature reduction and selection for emg signal classification. *Expert Systems with Applications* **39** (2012) 7420–7431. doi:10.1016/j.eswa.2012.01.102.

Breiman L. Random forests. *Machine Learning* **45** (2001) 5–32. doi:10.1023/A:1010933404324.

Dietterich TG. Ensemble methods in machine learning. Goos G, Hartmanis J, van Leeuwen J, editors, *Multiple Classifier Systems* (Berlin, Heidelberg: Springer Berlin Heidelberg), *Lecture Notes in Computer*

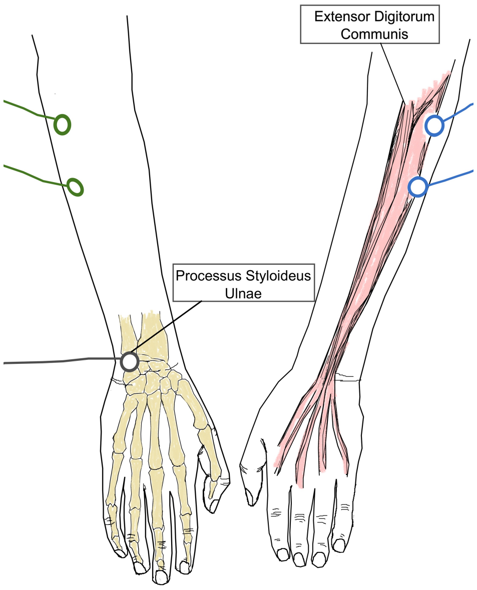

Figure S1. Electrode Placement for EMG recordings. EMG data was recorded from a bipolar channel (green and blue) placed on the left and right extensor digitorum communis muscles to control for finger movement in the offline analysis. In addition, a ground electrode (grey) was placed on the processus styloideus ulnae.

Science, vol. 1857 (2000), 1–15. doi:10.1007/3-540-45014-9{\textunderscore}1.

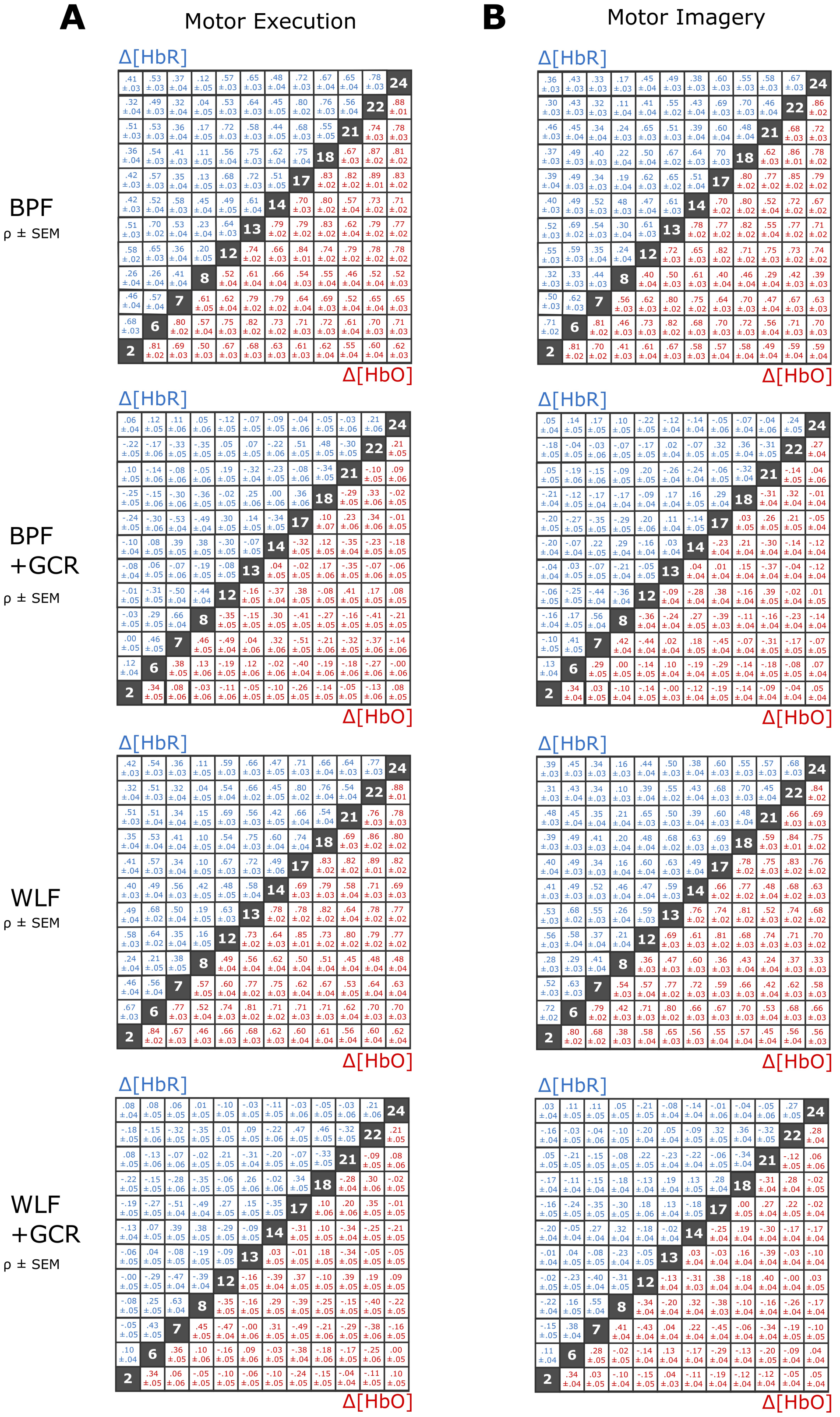

Figure S2. Mean ± SEM Spearman correlation coefficients (ρ) resulting from the spatial specificity approach (cf. sections 3.6.2 and 4.2). For the sake of completeness and in addition to the color coded correlation matrices in figures 6 and 7 all mean ± SEM ρ values between all contralateral channels of (A) motor execution and (B) motor imagery data. Each row represents one preprocessing approach of both $\triangle[HbO]$ (red; lower triangular matrix) and $\triangle[HbR]$ (blue; upper triangular matrix) data. The diagonal shows all contralateral channels.
